# Supplementary figures and images for: Use of virtual consultations in an orthopaedic rehabilitation setting: how do changes in the work of being a patient influence patient preferences? A systematic review and qualitative synthesis
Source: BMJ Open. 2020 Sep 16;10(9):e036197. doi: 10.1136/bmjopen-2019-036197 (PMC7497523; doi:10.1136/bmjopen-2019-036197)

Figure 1 – PRISMA Flow Diagram of included and excluded studies

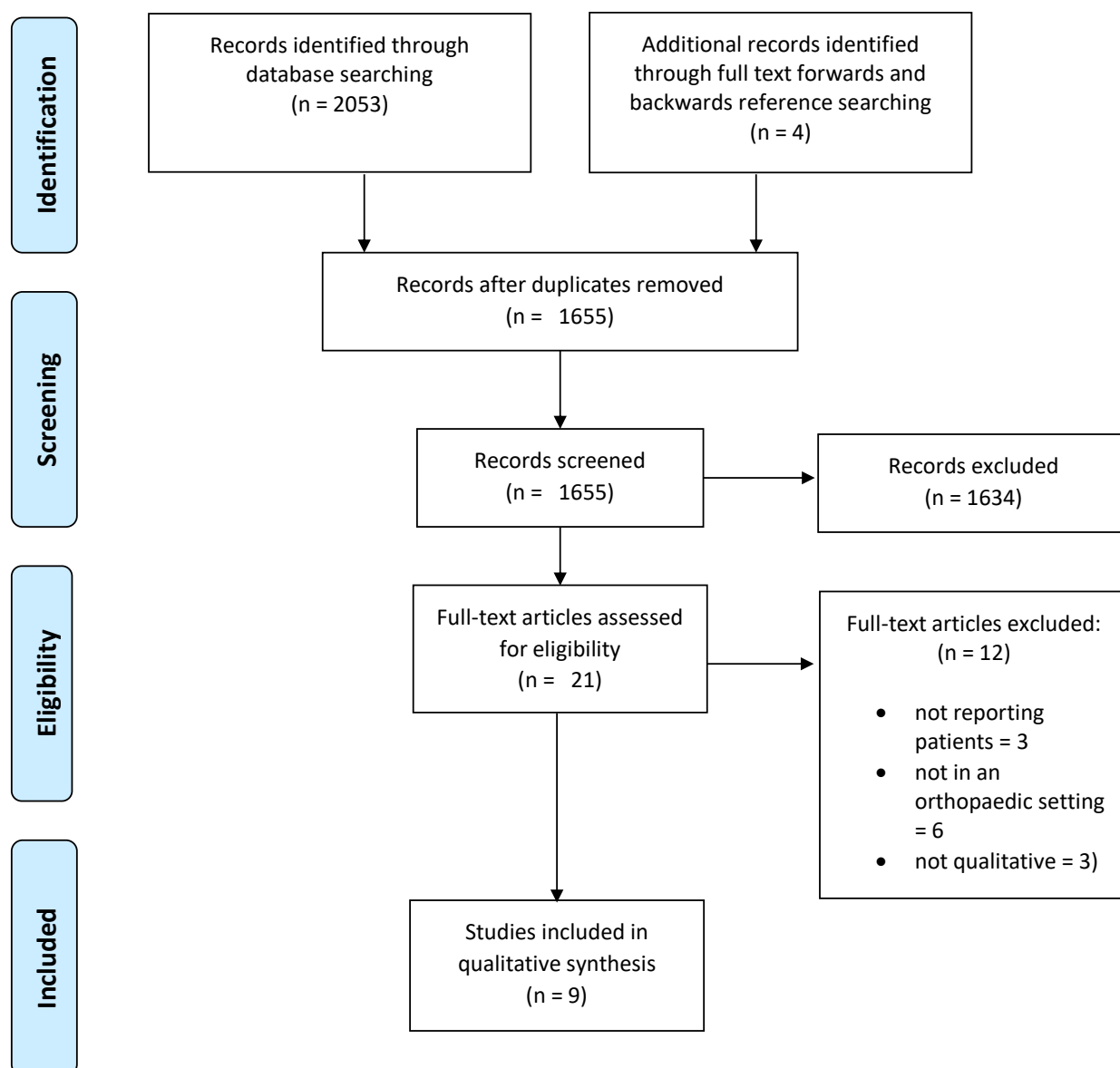

Supplement: Supplementary data [file bmjopen-2019-036197supp002.pdf]

Figure 2 – Visual Model to demonstrate how work influences preference.

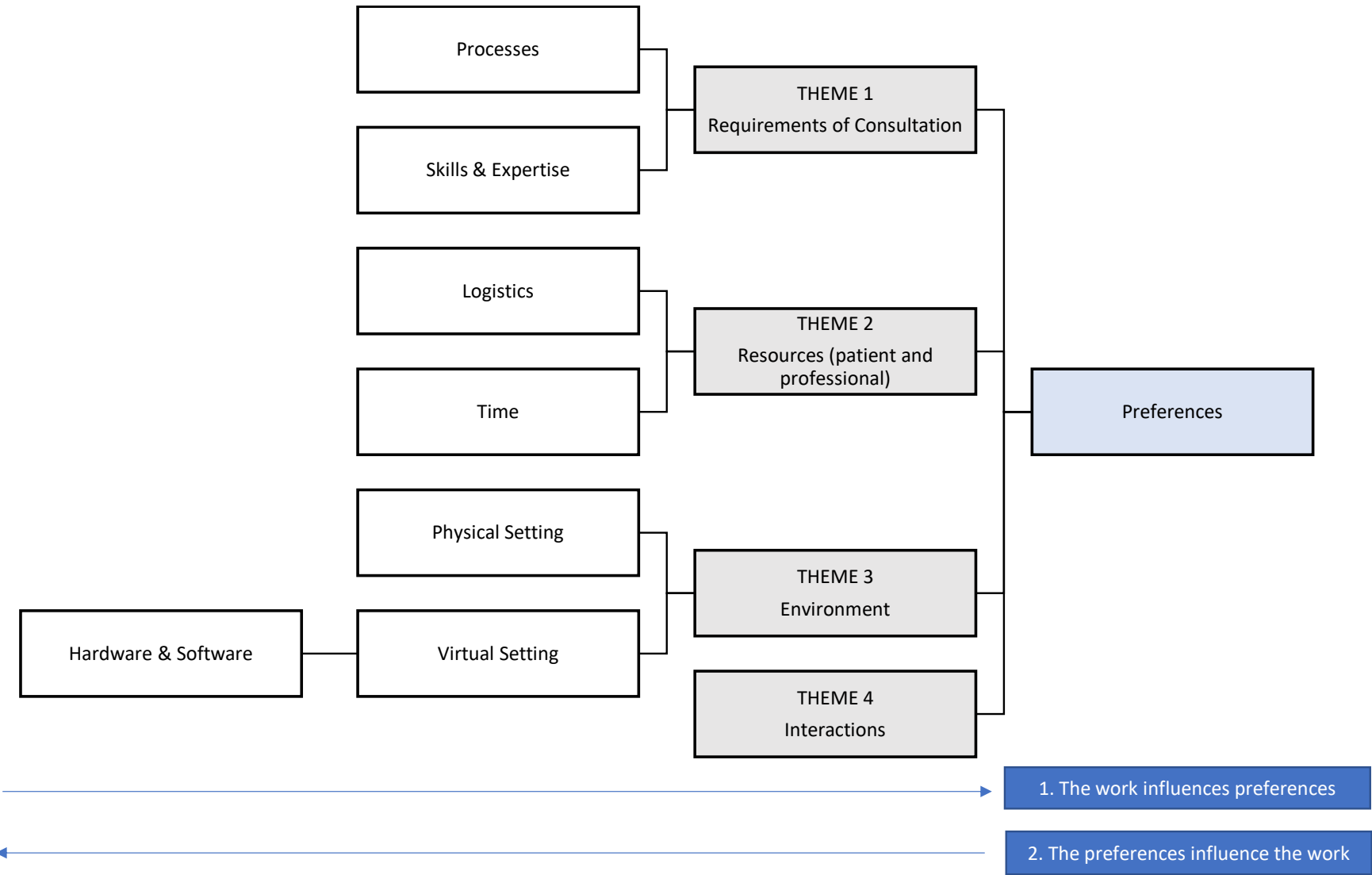

Supplement: Supplementary data [file bmjopen-2019-036197supp003.pdf]
