# Supplementary material for: Use of virtual consultations in an orthopaedic rehabilitation setting: how do changes in the work of being a patient influence patient preferences? A systematic review and qualitative synthesis
Source: BMJ Open. 2020 Sep 16;10(9):e036197. doi: 10.1136/bmjopen-2019-036197 (PMC7497523; doi:10.1136/bmjopen-2019-036197)
Supplement: Author's manuscript [file bmjopen-2019-036197.draft_revisions.pdf]

BMJ Open

BMJ Open is committed to open peer review. As part of this commitment we make the peer review history of every article we publish publicly available.

When an article is published we post the peer reviewers' comments and the authors' responses online. We also post the versions of the paper that were used during peer review. These are the versions that the peer review comments apply to.

The versions of the paper that follow are the versions that were submitted during the peer review process. They are not the versions of record or the final published versions. They should not be cited or distributed as the published version of this manuscript.

BMJ Open is an open access journal and the full, final, typeset and author-corrected version of record of the manuscript is available on our site with no access controls, subscription charges or pay-per-view fees (http://bmjopen.bmj.com).

If you have any questions on BMJ Open's open peer review process please email info.bmjopen@bmj.com

BMJ Open

How do changes in the 'work' of being a patient when using communication technology influence patient preferences? A systematic review and qualitative synthesis

Journal:	*BMJ Open*
Manuscript ID	bmjopen-2019-036197
Article Type:	Original research
Date Submitted by the Author:	04-Dec-2019

Complete List of Authors:	Gilbert, Anthony; Royal National Orthopaedic Hospital Stanmore, Therapies Department; University of Southampton, Faculty of Health Sciences<br>Jones, Jeremy; University of Southampton, Faculty of Health Sciences<br>Jaggi, Anju; Royal National Orthopaedic Hospital Stanmore, Therapies Department<br>May, Carl; London School of Hygiene and Tropical Medicine Faculty of Epidemiology and Population Health

Keywords:	Telemedicine < BIOTECHNOLOGY & BIOINFORMATICS, ORTHOPAEDIC & TRAUMA SURGERY, QUALITATIVE RESEARCH, REHABILITATION MEDICINE

SCHOLARONE™
Manuscripts

I, the Submitting Author has the right to grant and does grant on behalf of all authors of the Work (as defined in the below author licence), an exclusive licence and/or a non-exclusive licence for contributions from authors who are: i) UK Crown employees; ii) where BMJ has agreed a CC-BY licence shall apply, and/or iii) in accordance with the terms applicable for US Federal Government officers or employees acting as part of their official duties; on a worldwide, perpetual, irrevocable, royalty-free basis to BMJ Publishing Group Ltd ("BMJ") its licensees and where the relevant Journal is co-owned by BMJ to the co-owners of the Journal, to publish the Work in this journal and any other BMJ products and to exploit all rights, as set out in our licence.

The Submitting Author accepts and understands that any supply made under these terms is made by BMJ to the Submitting Author unless you are acting as an employee on behalf of your employer or a postgraduate student of an affiliated institution which is paying any applicable article publishing charge ("APC") for Open Access articles. Where the Submitting Author wishes to make the Work available on an Open Access basis (and intends to pay the relevant APC), the terms of reuse of such Open Access shall be governed by a Creative Commons licence – details of these licences and which Creative Commons licence will apply to this Work are set out in our licence referred to above.

Other than as permitted in any relevant BMJ Author's Self Archiving Policies, I confirm this Work has not been accepted for publication elsewhere, is not being considered for publication elsewhere and does not duplicate material already published. I confirm all authors consent to publication of this Work and authorise the granting of this licence.

For peer review only

How do changes in the 'work' of being a patient when using communication technology influence patient preferences? A systematic review and qualitative synthesis

Authors

1. Anthony Gilbert[1, 2]
2. Jeremy Jones[2]
3. Anju Jaggi[1]
4. Carl R May[3]

Is the order correct?

Author Affiliations

1. Therapies Department, Royal National Orthopaedic Hospital, Stanmore, UK

2. School of Health Sciences, Faculty of Environment and Life Sciences, University of Southampton, Southampton, UK

3. Faculty of Public Health and Policy, London School of Hygiene and Tropical Medicine, London, UK

Corresponding author: Anthony Gilbert anthony.gilbert@nhs.net

Abstract

Objectives

To systematically review qualitative studies reporting the use of communication technology within an orthopaedic rehabilitation setting.

Methods

Following the Preferred Reporting Items for Systematic Reviews and Meta-Analysis Statement we conducted a systematic review of papers to answer the research question 'How do changes in the 'work' of being a patient when using communication technology influence patient preferences?' Electronic databases were searched for studies meeting the inclusion criteria in July 2018.

Results

The search strategy identified 1596 research articles from the database search. A review of titles and abstracts using the inclusion criteria yielded 19 articles for full text review. Eight studies were included in the final analysis. Five studies explored real time videoconferencing and three explored telephone consultations. The use of communication technology changes the 'work' required of patients. Such changes will impact on expectations for care, resources required of patients, the environment of receiving care and patient-clinician interactions. This adjustment of the 'work' required of patients who access orthopaedic rehabilitation using communication technology will

For peer review only

impact on their experience of receiving care. It is proposed that changes in the ‘work’ of being a patient will influence preferences for or against the use of communication technology consultations for orthopaedic rehabilitation.

Conclusion

We found that the use of communication technology changes the ‘work’ of being a patient. The change in ‘work’ required of patients can be both burdensome (it makes it harder for patients to access their care) and beneficial (it makes it easier for patients to access their care). This change in ‘work’ is likely to influence preferences for use and we have demonstrated the importance of considering this ‘work’ when designing and implementing new technologies. Keeping the concept of patient ‘work’ at the heart of technology implementation is likely to be a key consideration to ensure successful implementation.

Registration

PROSPERO registration number: ID=CRD42018100896.

Strength and limitations of this study

- A taxonomy of patient ‘work’ will assist in understanding implementation processes.
- The use of middle range theory has been employed to guide theorization of the data.
- A secondary analysis of data has been employed to explain concepts which the authors had not originally intended.
- The date range of included studies (2005-2018) include a range of technologies including the use of bespoke software which may present different challenges to modern off the shelf software.

For peer review only

Introduction

<u>Background</u>

The NHS Long Term Plan[1], The United Kingdom's health service's plan to 'make the NHS fit for the future of patients', advocates digital-first primary care and envisions the use of e-consultations to become a new option for every patient. The document targets improved access to primary care services with 95% of GP patients to be offered e-consultation and other digital services in 2019[2]. Such initiatives may remove the inconvenience of cost and requirement to travel for patients.

Digital care may can support the management of patients with long term conditions such as musculoskeletal disease[3]. More than half of all people with symptomatic knee osteoarthritis are younger than 65 years of age[4]. It is likely that many of the patients with orthopaedic problems will live for another 2-3 decades during which time disability can occur. Long term management may require repeat visits for appointments with healthcare practitioners. Communication technology, the use of digital to support the communication from a distance, is a potential digital innovation that can enable patients to attend appointments. The PhysioDirect telephone and advice service[5] is an example that was found to be safe and resulted in equivalent physical outcomes to face to face appointments for patients with musculoskeletal disorders. The visual component offered with videoconferencing software offers distinct advantages over telephone consultations[6]. There are several examples of research investigating patients using Skype, a free to access videoconferencing software, to access care[7]. Patients who received telerehabilitation for knee arthritis via SKYPE[8] found it to be feasible and acceptable. The VOCAL study[9] found video outpatient consultations to be safe, effective and convenient in appropriate situations.

The process of implementing a new intervention (such as the introduction of communication technology in healthcare) has been demonstrated to be dependent on how the intervention is operationalised by its users[10], the 'work' people do when they implement a new intervention[11] and the mobilisation of resources over time[12] across different settings[13]. Normalisation Process Theory (NPT) frames implementation processes through its focus on the things people *do* when they implement a new intervention in practice. One study investigated nurse call takers conducting a physical assessment of patients' over a telephone helpline[14]. The study reported nurses' interactions with patients as they instructed them over the phone to perform physical manipulations. The accomplishment of a physical examination required 'work' from patients that differs to face to face consultations. Burden of treatment theory[15] explains how the capacity for action interacts with the 'work' that stems from healthcare. Burden of treatment has been demonstrated to arise when the workload demands exceeds the capacity for patients with COPD and lung cancer[16]. An understanding of the factors that contribute to a change in the 'work' for patients using communication technology is an important consideration for patient experience.

Research conducted in the UK found that the majority of people say they would use video consultations to consult their General Practitioner about minor ailments and ongoing condition[17]. A proportion (approximately 35%) would not use this modality. Our previous research investigated whether patients preferred face to face or virtual consultations[18]: patients with atraumatic shoulder instability were offered the choice between SKYPE and face to face follow up rehabilitation appointments. Half of patients preferred to see their rehabilitation professional in person[18] in part due to not having access and knowing how to use the software and equipment. The use of SKYPE changed the 'work' patients needed to do to engage in their care in our small study and this influenced their choice on whether or not to use it.

Preferences are a set of complex factors that may include enjoyment comparisons *(x to y is preferred if someone enjoys x more than they enjoy y)*, comparative evaluations *(x to y is preferred if someone thinks x is better than y)*, favouring *(selecting x over y because x has a particular set of characteristics)* or choice ranking *(x is chosen over y if and only if they are faced with a choice of x over y)*[19]. To get past the complexities of preferences, preferences can be defined as a 'total subjective comparative evaluation'[20]. In essence, someone will prefer x over y after consideration of the alternatives, the actions, the state of affairs and the consequences of choosing each alternative. In this paper we are interested in understanding how patient 'work' influences patient preferences.

Aims of this review

This paper reviews qualitative literature on the use of communication technology for patients in an orthopaedic rehabilitation setting to understand how the 'work' of being a patient influences preference. The purpose of this paper is to develop a taxonomy of tasks required of patients using communication technology. We then consider how factors relating to these tasks influence the comparative evaluation patients face when offered the choice of a communication technology or a face to face consultation for orthopaedic rehabilitation.

Methods

A systematic review was conducted using the PRISMA approach (See Supplementary Material 1 for reporting) in order to answer the research question: *How do changes in the 'work' of being a patient when using communication technology influence patient preferences?* This review was registered on the International prospective register of systematic reviews (PROSPERO registration number: ID=CRD42018100896)[21]. The protocol for the review, which forms phase 1 of the CONNECT Project, has previously been published[22].

MEDLINE, AMED, CINAHL, PsychINFO and SCOPUS were from inception on the 25th July 2018. Full search terms and the search strategy is available to view in Supplementary Material 2. Articles were screened independently by two authors (AG and AJ) with a third author (JJ) available to discuss any discrepancies.

Studies were eligible for inclusion providing they met the criteria for inclusion shown in Table 1. Relevant studies were firstly screened by their title and then by their abstract. Remaining texts were then read in full with all texts retained after this point for qualitative synthesis. Risk of bias was screened using the CASP tool for qualitative studies[23]. A discussion was held, between two authors (AG and AJ) with a third author (JJ) available to discuss any discrepancies, to decide whether included studies were of sufficient quality to include in the review.

For peer review only

Table 1: Eligibility Criteria of Studies

Inclusion:	Exclusion:
• Full text English Language academic papers from inception to 25th July 2018.<br>• Patients with an orthopaedic / musculoskeletal problem<br>• Studies reporting patients accessing physical assessment / rehabilitation through the use of communication technology (e.g. telephone, videoconferencing) in an orthopaedic / musculoskeletal setting.<br>• Qualitative studies or studies with a qualitative component that focuses on the patient viewpoint of accessing communication technology.	• Conference abstracts<br>• Participants without an orthopaedic / musculoskeletal complaint<br>• Quantitative studies<br>• Studies not reporting patient viewpoints

Full texts were uploaded to QSR NVIVO Software (QSR International Pty Ltd. Version 12, 2018). NVIVO was used to collect and organize data from the results, discussion and conclusion sections of each paper. Each sentence from the included sections were coded on a line by line basis. The codes were labelled using a description of the content of the respective sentence. Data analysis subsequently took three forms: firstly, two authors (AG and CM) conducted a thematic analysis of codes. This was undertaken to familiarize the authors with the content of the papers. For the second iteration of coding the following was considered: *what is the 'work' of being a patient when using communication technology?* Codes were then organized into groups depicting the *type* of 'work' required of patients when using communication technology to access healthcare. The two authors (AG and CM) then considered the question: *how might the 'work' of being a patient when using communication technology influence patient preference?* The data were revisited and theoretical ideas arising from the data were discussed between AG & CM. From here themes, empirical regularities in the data, were identified and characterised. Finally, themes arising from the data were mapped out in the form of a model to demonstrate how, based on the included papers, the change in the 'work' of being a patient might influence preference for communication technology.

For peer review only

Figure 1 – PRISMA Flow Diagram of included and excluded studies

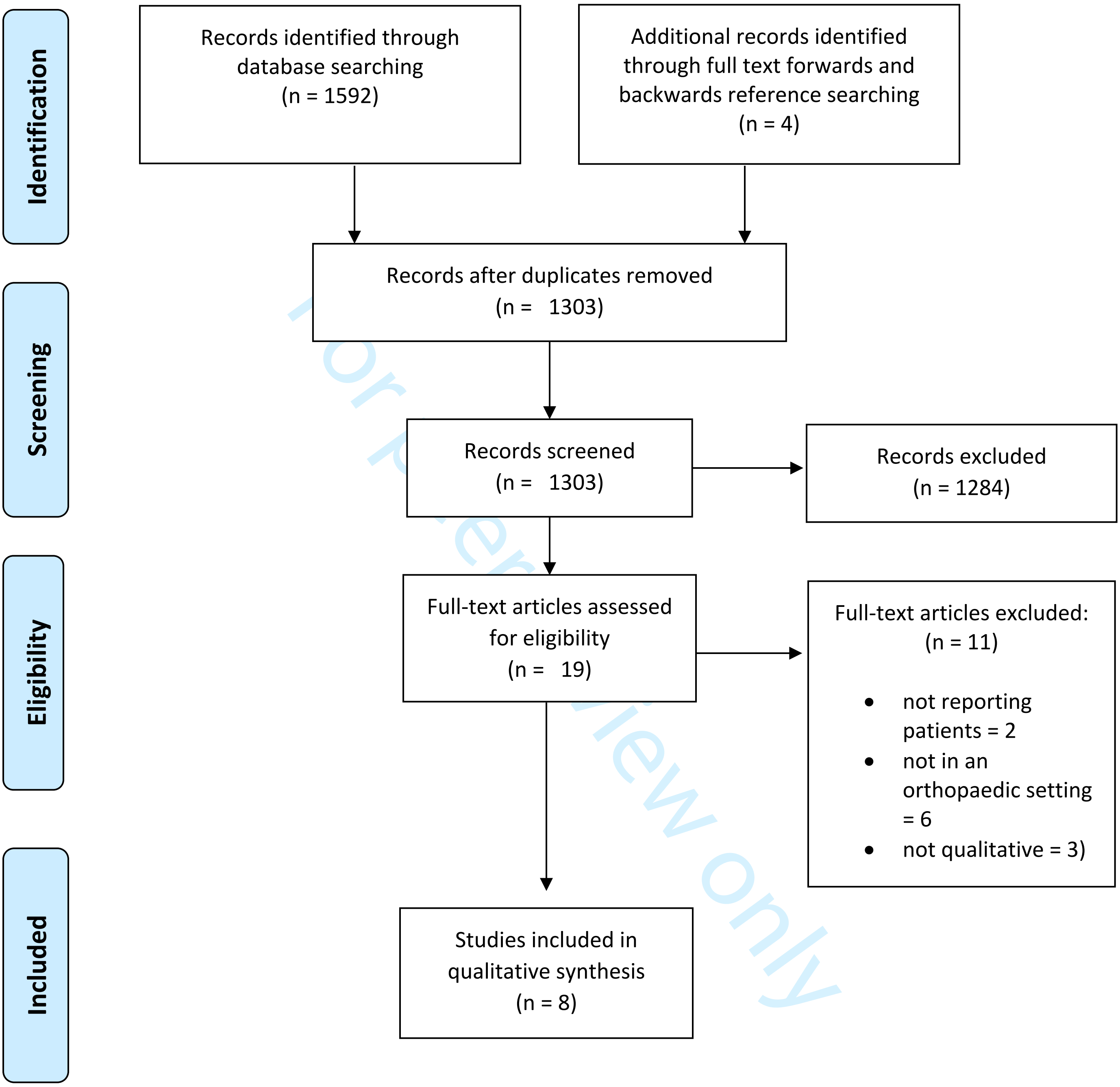

Table 2 – Study Characteristics.

Included Study	Study Setting	Study Purpose	Technology Used	Participants
Harrison et al 2006[24]	Joint teleconsultations between the patient and their GP and a hospital specialist (England)	To explore patients' experiences of joint teleconferenced consultations	ISDN2 link and off-the-shelf videoconferencing software	28 patients who were enrolled in the Virtual Outreach Randomized Trial[25]. Six patients had a generic orthopaedic diagnosis.
Young et al 2007[26]	Telephone and videophone follow up after scoliosis surgery (Canada)	To better understand the relative effectiveness of two types of telehealth technology, telephone versus videophone, following a child's scoliosis surgery from the perspective of patients and care-givers	For the videophone group patients were provided with a videophone (KXC-AP150, Panasonic, Japan). For the telephone group patients used an ordinary telephone line	43 patients and their families (dyads) who had undergone scoliosis correction surgery. 21 dyads received videophone support and 22 dyads who received telephone support.
Eriksson et al 2011[27]	Video-based physiotherapy at the patient's home for two months after a shoulder replacement (Sweden)	To describe patients' experiences of physiotherapy at home by video-link after a shoulder replacement	Standard commercial videoconferencing units (e.g. Tandberg 800, Sony PCS-50, Polycom VSX 3000)	10 Adults who had undergone a shoulder replacement.
Cranen et al 2011[28]	Telerehabilitation services at a rehabilitation centre (Netherlands)	To explore patients perceptions regarding prospective rehabilitation services and the factors that facilitate or impede patients' intentions to use these services	Home based treatment by means of (unspecified) web cam treatments	25 chronic pain patients from a rehabilitation centre.
Kairy et al 2013[29]	Telerehabilitation between the patient at home and the physical therapist at the hospital (Canada)	To better understand the patient's experience of home telerehabilitation	Internet access and the telerehabilitation platform was installed in the patient's home as reported in[29].	5 patients who had previously received in-home telerehabilitation post knee arthroplasty. Patients were

For peer review only

			The telerehabilitation device was custom built for the study	selected from a pool of participants from the experimental arm of a RCT for in-home telerehabilitation[30].
Pearson et al 2016[31]	Telephone based physiotherapy between a patient and a senior physiotherapist (England)	To describe key variables that determined patient acceptability of the PhysioDirect service and to understand how the patient experience differed from those accessing usual physiotherapy care.	Telephone	57 patients with a musculoskeletal problem. Participants were recruited from the PhysioDirect study[32].
Hinman et al 2017[8]	SKYPE mediated physiotherapy consultations between the patient at home and the physiotherapist (Australia)	To explore the experience of patients and physical therapists with Skype for exercise management of knee osteoarthritis (OA)	SKYPE software	12 patients with a diagnosis of knee osteoarthritis. Participants were key informants from an RCT[33].
Lawford et al 2018[34]	Exercise therapy for people with knee arthritis via telephone (Australia)	To explore people's perceptions of exercise therapy delivered by physiotherapists via telephone.	Telephone	20 patients with knee osteoarthritis. Participants with knee OA were recruited as key informants from an RCT[35].

For peer review only

Results

Study Selection

Systematic search identified 1,303 references (after de-duplication) of which 1,284 were excluded on the basis of titles and abstracts and a further 11 excluded at full text review. As a result 8 papers were included in the review. Of the eight papers, two originated from Australia[8 34], 2 from Canada[26 29] and 2 two from England[24 31] and with one from Sweden[27] and one from the Netherlands[28]. Five studies explored real time videoconferencing[8 24 26-29] and three explored telephone consultations[26 31 34]. Study demographics are shown in Table 2. All studies were screened using the CASP tool for qualitative studies[27] and all were deemed by the authors to be of sufficient quality and therefore retained for analysis.

Worked Example of Data Analysis

Data from the eight studies were synthesized. All data were treated to the same three step process. An exemplar is demonstrated below using data from Eriksson et al[27]:

1. Data identified (initial line by line identification)

Inability to touch the patient meant therapists were forced to rely more on their subjective assessment of the patient, leading them to spend more time talking with and listening to patients.

2. Data characterized (initial line by line coding)

Code assigned: *Therapists were unable to use 'hands on' during assessment.*

3. Data theorized (consideration of the question: *what is the 'work' of being a patient when using communication technology?*)

Patients have to present themselves in a different way during assessment via VC.

Synthesis of Results

Theme 1: Expectations of rehabilitation

1.1 The skills that change

The use of communication technology changed the skills patients needed. Video communication required specific communication skills that included listening with close attention with no interruptions[27]. The gaze of the patients and clinicians were used to signal the start and end of conversations[27]. Patients and their families found it challenging to express how they felt from a distance and were reliant on the visual capabilities of the technology[26]: *'I think at times when we had trouble expressing the right words, how you feel and where exactly it hurts… there's nothing like showing everything… a picture speaks a hundred words.'* The lack of visual information was a concern for patients in the PhysioDirect service[31] who did not have visual cues and physical contact. The lack of physical contact meant that therapists were more reliant on information shared by patients rather than those derived from physical tasks[8]. Therapist focussed on more effortful treatments such as exercises and self-management rather than providing them with hands on care[8].

1.2 The processes that change

For peer review only

The use of communication technology required additional steps for patients, such as logging in[28] and setting the software up[8]. Patients also needed to obtain equipment, normally available in the clinic, to use in the community[29]. Some patients valued the portability of using Skype[8] and found that they could use it across different settings to fulfil the purposes of the consultation.

1.3 The expertise that is required

Traditional face-to-face interaction is well established and accepted. It was recognised that virtual communication required different skills and therapists' training needs, to ensure effective communication with patients, were considered in one study of telephone consultations[34]. Traditional physiotherapy patient assessment (such as 'hands on' palpation of a joint) is not possible via Skype. As a result of this patients were taught to self-palpate under guidance[8] and instructed how to demonstrate their range of movement over the screen. It is self-evident that visual assessment was not possible over telephone[26 31 34] and this required good communication from both therapists and patients to describe the movements. Patients felt they did not need 'hands on' care when they were seen by an experienced therapist[27] and clinicians were more likely to encourage self-management and exercises when they were seen virtually[31].

Theme 2: Resources

2.1 Logistics

Patients who underwent virtual consultations experienced reduced travel times and transportation issues[8 27-29 34] and was often seen as more convenient for patients, particularly those who suffered from chronic pain[8]. Virtual consultations enabled patients to access health services more easily[24 29]. Problems did arise with the PhysioDirect service where patients were unable to get through requiring them having to make multiple calls to speak to a therapist.

2.2 Time

Virtual consultations offered flexibility[34]: *'If I know I'm stuck at work and I can't get to see someone [the telephone] would be a good option…I can ring someone or have an appointment on the phone, and be at work doing what I need to do, and still have my appointment.'* It was particularly useful for patients who had multiple commitments: *'Because life's so busy in general too, so to be able to speak to somebody in your home and then you can go on with your, you know, your next thing, is just wonderful…it just opens another brilliant option for people'* as it provided more time for other activities and to integrate rehabilitation into daily life[27].

Theme 3: Environment

3.1 Setting for rehabilitation

Rehabilitation in the home was welcomed by some patients as it gave them the opportunity to rehab within their own environment whereas other patients preferred to keep their home environment separate from the clinical environment[28]. Patients found that they had a lack of space at home compared to the clinic[8 28] and could not access clinic-based equipment[8 29]. Rehabilitation required patients to troubleshoot ways to integrate their rehabilitation tasks within the home[8 34].

3.2 Setting for virtual consultation

Some patients valued fellow sufferer contact and felt that through not physically attending the clinic they missed out on stimuli which kept them motivated. Rehabilitation was impaired when there were issues with connectivity and audio-visual interference disrupted the flow of the consultation[8].

For peer review only

Some patients felt that telerehabilitation was as good as real life and didn't affect the flow of the consultation[27].

3.3 Hardware and Software

Patients who did not have access to equipment for virtual consultation needed to be provided with the required hardware[26 27 29]. In some cases, significant support was required for patients to understand how to use the equipment [28 8 26] and to troubleshoot connection problems when they arose[8 26 27 29]. Overcoming these barriers was an important factor in maintaining the quality of the virtual consultation and is likely to require technical support provided by the clinical team[8].

Theme 4: Interactions

Some patients reported being more relaxed in their own home[8]. One patient, however, felt uncertain about having someone looking into their home and aborted the video consultation[27]. Virtual interactions were impaired at times there was a poor connection[8 27] or a language barrier[26]. These situations demanded additional focus and non-verbal communication[24] from the patient. The therapeutic relationship between patients and clinicians is negatively affected when patients feel alienated[28] or detached[28 31] from their clinician. Patients with an expectation of hands on care[8 26 31] found virtual rehabilitation more challenging and may need to invest additional effort to maintain an effective relationship with their therapist.

For peer review only

Table 3 - Factors that may affect patient preference for communication technology consultations

**Finding**	**Construct**	**Results from included papers: factors that contribute towards the 'work' of being a patient when using communication technology**
Preferences are shaped by the expectations of how the consultation changes the 'work'	The skills that change	As patients moved away from physically facilitated exercises there was the requirement to adjust[27], overcome patient-clinician communication difficulties over video-call[24 26] and phone call[31] and face an increased reliance on them to communicate information[8]. In the absence of hands on treatment more emphasis is placed on patients completing exercises[8].
	The processes that change	Patients were able to engage in consultation from different places[8]. Using communication technology required patients to arrange for additional equipment in the home[29]. They were required to log in to an account[28] and to learn how to use the communication technology[8].
	The expertise that is required	Patients need to self-assess when they cannot be physically assessed by a therapist[8]. Patients may need to adapt to clinicians who do not have adequate communication skills or training for using communication technology[34]. Patients may be encouraged to self-monitor improvements more than if they were seen face to face[31].
Preferences are shaped by the resources that are required of patients	Logistics	Use of communication technology helps to avoid of transportation issues[8 27-29 34], reduces travel times[28] for both patients and carers and can increase access to services[8].
	Time	The ease in which exercises can be integrated into home routine[28] and through avoidance of travel provides additional time and energy for other activities[27]. Patients valued being able to wait for their appointment in their own chosen environment rather than in the clinic[8 24 31].
Preferences are shaped by the 'work' required due to the changes in the environment	Setting for physical rehabilitation	Patients had to find ways to overcome a lack of space[8 28] and equipment[8 29] at home. Patients were required to integrate their rehabilitation in the home environment[8 34].
	Setting for virtual consultation	At times the rehab was impaired due to technical difficulties[8] and patients felt they missed learning through fellow sufferer contact through not attending the clinic and would need to seek this elsewhere[28].
	Hardware and software	Patients needed to be supported to access[8 29] and use the equipment[8 26 28] and manage to real-time troubleshoot connection problems as they arose[8 26 27 29].
Preferences are shaped by the 'work' that goes into maintaining adequate interactions	Interactions	Patients may have to focus additional attention when communicating over a stutter connection[8 27] or when faced with a language barrier[26]. Patients may need to rely on additional non-verbal communication when communication over a screen[24]. Patients who feel alienated[28] or detached[28 31] or expect hands on care[8 26 31] may need to invest additional effort in developing an effective therapeutic the patient clinician relationship.

For peer review only

Figure 2 – Visual Model to demonstrate how ‘work’ influences preference.

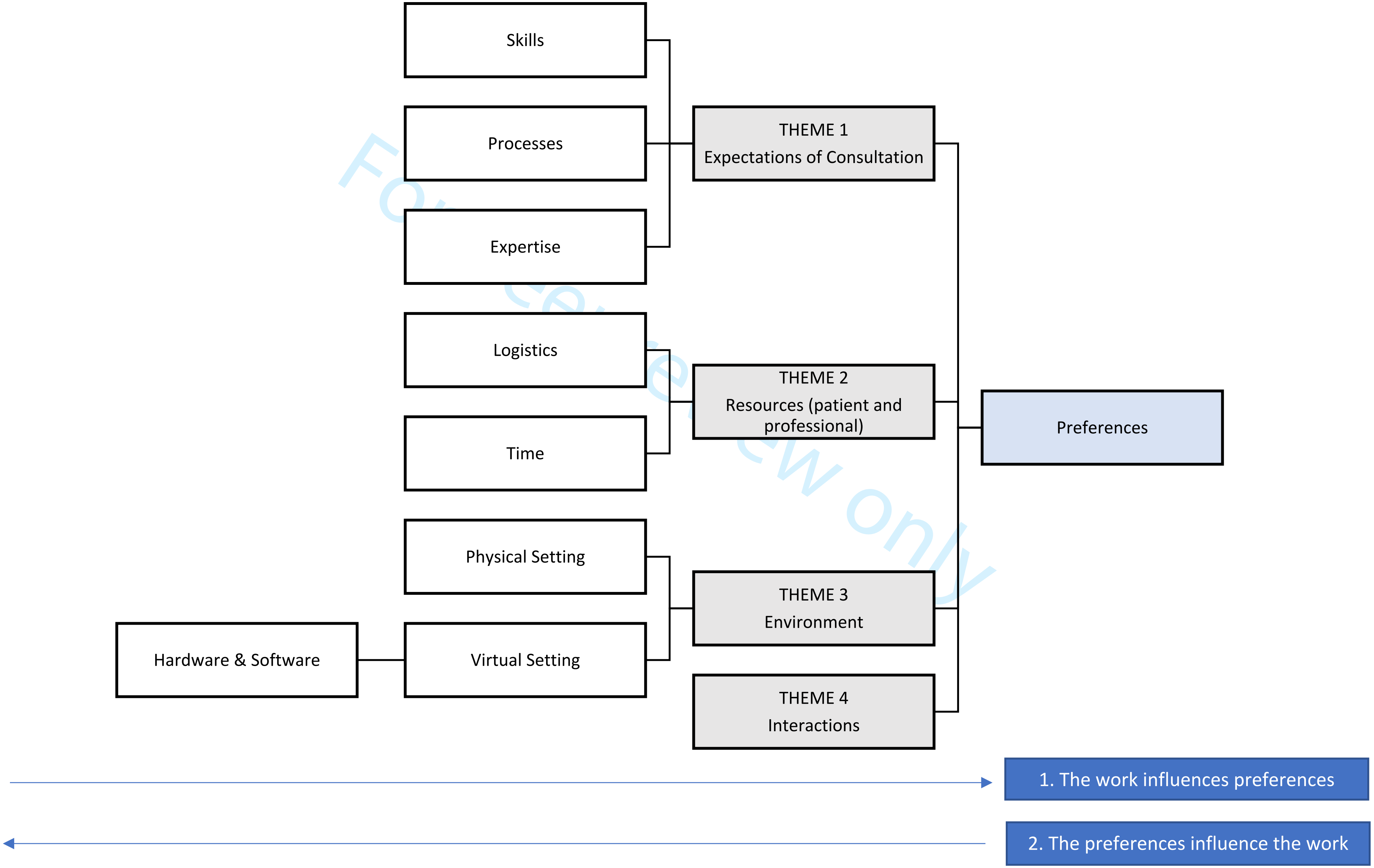

Discussion

This review synthesized eight qualitative studies reporting the use of communication technology in an orthopedic setting. We explored how the use of these technologies impacts on the 'work' of being a patient. All studies in this review demonstrated that adjustments are required of patients to operationalise communication technology for clinical interactions. The adjustments (in the 'work') that a patient needs to make will have an effect on their experiences of receiving care. These experiences, whether previously lived or anticipated in the future, are likely to influence whether or not an individual finds the use of communication technology acceptable. The patient preference for a communication technology consultation will depend on individual circumstances. Some of these factors which might influence their decision have been and presented in a conceptual model. The model attempts to demonstrate the relationship between patient 'work' and preference when using communication technology. The model suggests that the use of communication technologies changes the 'work' of being a patient. The consequences (both positive and negative) of these alterations in 'work' may impact on the patient's experience of receiving their healthcare, their burden of treatment and their ability to engage with their healthcare. This is an important consideration for clinician, managers and policy makers.

Clinicians have to pay more attention to the patient as a result of communicating using technology compared to face to face consultations[9]. This appeared to be at odds with traditional consultations where physiotherapists spoke for half of the allotted time compared to patients who spoke for only 33.1%[36] in initial encounters. A study found, during a follow up session between physiotherapists and patients, that physiotherapists spent twice as much time talking as the patients did and they relied on the use of their hands during the session[37]. In addition to the content within sessions, the relationship experienced between the clinician and the patient may differ during a virtual consultation due some patients being more relaxed at home[9].

Some patients expected 'hands-on' treatment. The transfer of clinician manual therapy towards patient self-palpation[8] and exercise[31] may go against what is expected of therapists. The normative expectations of the patients change as a result of the geographical separation (and physical resources that can be mobilized) between patient and therapist[38]. This places particular emphasis on self-management which shifts the responsibility for health away from the state and onto the individual[39]. The additional responsibility of self-management[40], the change in 'work' and tasks required to operationalise communication technology may further burden patients as they are rehabilitated virtually.

Patient viewpoints are important. Kaambwa et al[41] found in their study of older people that patients had strong preference for telehealth services that targeted individuals living in remote regions without easy access to clinic. Our previous research[18] demonstrated that distance to travel to a hospital was not the sole reason leading to the acceptability of Skype consultations and that preference is multi-factorial. We found that having rehabilitation in the patient's own environment was preferred by some although bringing the clinical space into the patient's home can change the meaning of their home for them[42]. Greenhalgh et al[43] considers, amongst other things, *what is expected* of the patient when using new technologies and explains that complex tasks are more likely to lead to non-adoption.

Greenhalgh et al's VOCAL study[9] found that the situations where patients were appropriate for video outpatient consultations only formed a fraction of the overall workload. Such situations included when close physical examination was not required and when both parties were technically confident

and competent. The use of communication technology in these situations may increase patient 'work' and therefore contribute towards their burden of treatment. Patients may therefore opt to choose a face to face consultation. Sav et al[44] call for collaborative discussions to help alleviate treatment burden.

Consider these two hypothetical situations; imagine both of these patients have a shoulder problem and have been offered the choice between a face to face and a video-consult for their next session.

Table 4 - Practical application of results

Sarah	Paula
• *She lives one hour away from the clinic where she required physio*<br>• *She needs to take a half day off work to physically attend her appointment. She can use part of her lunch break for a virtual appointment*<br>• *She works full time*<br>• *She has two primary school age children*<br>• *Her spouse is currently working away and she does not have anyone that can pick the children up for her*<br>• *She is familiar with her exercises and has the equipment she needs to do at home*<br>• *She is familiar with the use of Skype and uses it regularly on her phone for business*<br>• *The rehab clinic she goes to routinely uses virtual clinics and evaluation demonstrates they are safe and effective*<br>• *Sarah's shoulder rehab is going well and this session is scheduled to be an 8 week 'checkup' to see how things are going*	• *She works 5-minute walk away from the clinic where she requires physio.*<br>• *She is self-employed and can be flexible with timings without the need to take holiday*<br>• *She works full time*<br>• *She has two primary school age children*<br>• *Her spouse works from home and is able to pick up the children*<br>• *Her last lot of exercises is causing her severe neck pain and she is concerned about the affect these exercises are having*<br>• *She has 'techno phobia' and is unfamiliar with any form of videoconferencing*<br>• *The rehab clinics are offering this new method as a pilot to test its feasibility*<br>• *Paula is unhappy with how her rehab is going and is concerned about her new neck pain. She wants the therapist to review and change her exercises.*

Sarah already has access to the equipment and software required and is familiar with its use. Using technology in this situation would save her taking half a day away from her employment and will remove travel time and childcare challenges. Sarah is happy with her rehab and only requires a simple checkup. Sarah's situation is different to Paula who is not comfortable with the idea of using technology, she can easily get to the clinic from her workplace and has flexibility to do so without the worry of having to organize childcare. It is apparent a virtual consultation would be more burdensome for Paula and less burdensome for Sarah.

There may be situations where preferences may change. Let's consider Sarah: imagine her shoulder problem got worse and required a physical review. The problem might interfere with her hobbies or employment and the therapist might suggest that a physical appointment would be worthwhile. As the situation changes the preference might change. Sarah might need to seek support from her

For peer review only

social network or to seek time away from her job to enable her to attend the appointment. She may need to mobilise additional resources to give the capacity to attend her appointment in order to make physical attendance less burdensome. As we have shown, treatment burden and preferences may be dynamic and change over time.

Digitally enabled services are a key focus for the UK's National Health Service over the next 10 years[1]. This may be useful for some but add to the burden of treatment to others. Tools have been developed to assess burden of treatment[45-49]. Further research investigating the utility of tools such as these may highlight areas where digitally enabled services negatively (or positively) impact on patient experience. Further research investigating patient preference will help researchers and clinicians tailor services in a way that suits the need of patients.

Figure 2 demonstrates how the themes from this review interact with patient preferences. The 'work' required of a patient will influence their expectations of whether or not the use of communication technology is acceptable. The logistics and time required of a patient will shape the resources the patient has to dedicate towards their care. The space available and the equipment the patient has access to determines the suitability of the environment. These, coupled with the impact on patient-clinician interactions will determine patient preference for or against communication technology. This leads us to our first preposition: Proposition 1: The 'work' of being a patient when using communication technology will influence their preferences

Face to face consultations and communication technology consultations have different requirements. Upon choosing a face to face consultation the patient follows the standard pathway. Choosing a communication technology consultation changes what is needed of patients. The change of work demands different skills, processes, expertise, logistical and environmental considerations. This in turn impacts on the nature of the interactions between the patient and their therapist. This leads us to our second preposition: Proposition 2: The preferences regarding the use of communication technology will influence the 'work' of being a patient.

Limitations of this review

Our review is subject to a number of important limitations. We included papers from the UK, Sweden, USA, Canada and Australia which utilized a variety of communication technologies. The data that underpins our results are a secondary analysis of other previously collected data. We did not have access to the original qualitative datasets, only that presented in the research papers. To arrive at our conclusions, we have subjected the data from the primary studies to explanatory concepts that the original authors had not intended. The studies spread from 2005 to 2018. During this time technology has advanced considerably and the bespoke software used in the earlier studies (that were developed for the research study) may present different challenges to modern off the shelf software for use with commonly used personal devices such as phones, tablets or computers. It is also important to acknowledge the differences between the different types of technologies. A phone call does not allow for visualization whereas a video call does. Focusing on specific technologies may have generated more applicable results. The original research recruited patients who had opted into these studies. Patients who are satisfied with these technologies are more likely to be recruited to telemedicine studies and may not be a representative sample.

Conclusion

We reviewed eight qualitative studies which reported the use of phone or videocall in orthopaedic care and found that the use of communication technology changes the work of being a patient. We

For peer review only

identified four different kinds of 'work' relating to: (1) the consultation, (2) the use of resources, (3) changes in the environment and (4) interactions with the healthcare professional. Across all four domains, the change in work required of patients can be both burdensome (it makes it harder for patients to access their care) and beneficial (it makes it easier for patients to access their care). The burden experienced by patients is a result of the relationship between the demands of the work and their capacity to fulfill these demands. Such burden is individual and situational, depending on the clinical requirements and the patient's lifeworld. As a result, we have proposed that the work of being a patient influences their preferences and the resulting choice has consequences on the resulting work that is required of them. Changes in circumstances (such as availability of equipment, understanding of how to use the equipment, requirements of the rehabilitation) may alter what is required both clinically and technologically and influence preferences. This is an important consideration to patients, clinicians, managers and policy makers, especially at a time where the use of technology is being internationally favored. We have demonstrated the importance of considering the work of being a patient when designing and implementing new technologies. Keeping the concept of patient work at the heart of technology implementation is essential to ensure successful uptake in practice.

Patient and Public Involvement

The CONNECT Project Patient and Public Involvement steering group (PPISG) has been set up to provide guidance on the conduct of the research (details available from www.theconnectproject.info). The first meeting of the PPISG was held in August 2016 prior to the submission of the research to the NIHR in May 2017. A discussion was held about the overall research aims which supported the identification of the research questions. The PPISG has supported the design of the overall research plan and will continue to be involved during the development and refinement of each phase prior to the completion of each study protocol. In addition, the PPISG will support the development of the lay-summary outputs to be disseminated to patients and members of the public. Links to research outputs will be made available on the CONNECT Project website available at www.theconnectproject.info.

Acknowledgements

The authors thank Professor Maria Stokes and members of the CONNECT Project Patient and Public Involvement steering group for their invaluable contributions to the overall study design of the CONNECT Project and obtaining funding for the PhD Fellowship. The authors also thank colleagues within the Therapies Directorate and Research and Innovation Centre at the Royal National Orthopaedic Hospital for their ongoing support.

Contributors

AG wrote the paper and conceived the project with CM and JJ. CM contributed knowledge on systematic reviews and qualitative analysis. AG and AJ completed the literature search, identification of papers and quality analysis of papers. CM, JJ, and AJ edited and critically revised the paper. All authors have read and approved the manuscript. AG is the guarantor of the manuscript.

Funding Statement

Anthony Gilbert is funded by a National Institute for Health Research (NIHR), Clinical Doctoral Research Fellowship for this research project (ICA-CDRF-2017-03-025).

For peer review only

Disclaimer

This paper presents independent research funded by the National Institute for Health Research (NIHR). The views expressed are those of the authors and not necessarily those of the NHS, the NIHR or the Department of Health and Social Care.

Competing Interests

Nil.

Patient Consent for Publication

Not required.

For peer review only

References

1. NHS. The NHS Long Term Plan. 2019. https://www.longtermplan.nhs.uk/wp-content/uploads/2019/01/nhs-long-term-plan-june-2019.pdf (Accessed 3rd October 2019).

2. Department of Health and Social Care. 2019. NHS Mandate 2018 to 2019 2018 [Available from: https://assets.publishing.service.gov.uk/government/uploads/system/uploads/attachment_data/file/803111/revised-mandate-to-nhs-england-2018-to-2019.pdf accessed 18th August 2019 2019.

3. WHO. Musculoskeletal conditions fact sheet [Available from: https://www.who.int/mediacentre/factsheets/musculoskeletal/en/ accessed 13th January 2019

4. Deshpande BR, Katz JN, Solomon DH, et al. Number of Persons With Symptomatic Knee Osteoarthritis in the US: Impact of Race and Ethnicity, Age, Sex, and Obesity. *Arthritis Care and Research* 2016;68(12):1743-50. doi: 10.1002/acr.22897

5. Salisbury C, Montgomery A, Hollinghurst S, et al. Effectiveness of PhysioDirect telephone assessment and advice services for patients with musculoskeletal problems: pragmatic randomised controlled trial. *BMJ: British Medical Journal* 2013;346(7893):14. doi: 10.1136/bmj.f43

6. Donaghy E, Hammersley V, Bikker A, et al. Acceptability, benefits, and challenges of video consulting: A qualitative study in primary care. *British Journal of General Practice* 2019;69(686):E586-E94. doi: 10.3399/bjgp19X704141

7. Armfield NR, Bradford M, Bradford NK. The clinical use of Skype—For which patients, with which problems and in which settings? A snapshot review of the literature. *International Journal of Medical Informatics* 2015;84(10):737-42. doi: 10.1016/j.ijmedinf.2015.06.006

8. Hinman RS, Nelligan RK, Bennell KL, et al. "Sounds a Bit Crazy, But It Was Almost More Personal:" A Qualitative Study of Patient and Clinician Experiences of Physical Therapist-Prescribed Exercise For Knee Osteoarthritis Via Skype. *Arthritis Care & Research* 2017;69(12):1834-44. doi: 10.1002/acr.23218

9. Vijayaraghavan S, Morris J, Bhattacharya S, et al. Real-world implementation of video outpatient consultations at macro, meso, and micro levels: Mixed-method study. *Journal of Medical Internet Research* 2018;20(4) doi: 10.2196/jmir.9897

10. May C. A rational model for assessing and evaluating complex interventions in health care. *BMC Health Services Research*. 2006;6. doi: 10.1186/1472-6963-6-86

11. May C, Finch, T. Implementing, Embedding, and Integrating Practices: An Outline of Normalization Process Theory. *Sociology* 2009;43(3):535. doi: 10.1177/0038038509103208

12. May C. Towards a general theory of implementation. *Implementation Science* 2013;8(1) doi: 10.1186/1748-5908-8-18

13. May CR, Johnson M, Finch T. Implementation, context and complexity. *Implementation Science.* 2016;11. doi: 10.1186/s13012-016-0506-3

14. Lopriore S, LeCouteur A, Ekberg K, et al. "You'll have to be my eyes and ears": A conversation analytic study of physical examination on a health helpline, 2019:330-39. doi: 10.1111/jocn.14638

15. May CR, Eton DT, Boehmer K, et al. Rethinking the patient: using Burden of Treatment Theory to understand the changing dynamics of illness. *BMC Health Services Research* 2014;14:281-81. doi: 10.1186/1472-6963-14-281

16. Lippiett KA, Richardson A, Myall M, et al. Patients and informal caregivers' experiences of burden of treatment in lung cancer and chronic obstructive pulmonary disease (COPD): a systematic review and synthesis of qualitative research. *BMJ Open* 2019;9(2):e020515-e15. doi: 10.1136/bmjopen-2017-020515

For peer review only

17. Castle-Clarke S. What will new technology mean for the NHS and its patients? Four big technological trends 2018 [Available from: https://www.nuffieldtrust.org.uk/files/2018-06/1530028974_the-nhs-at-70-what-will-new-technology-mean-for-the-nhs-and-its-patients.pdf.
18. Gilbert AW, Jaggi A, May CR. What is the acceptability of real time 1:1 videoconferencing between clinicians and patients for a follow-up consultation for multi-directional shoulder instability? *Shoulder & Elbow* 2019;11(1):53-59. doi: 10.1177/1758573218796815
19. Hausman DM. Preference, Value, Choice, and Welfare: Cambridge University Press 2012.
20. Hausman DM. Mistakes about Preferences in the Social Sciences, *Philosophy of the Social Sciences.* 2011:3-25. doi: 10.1177/0048393110387885
21. Gilbert AW, Jaggi C, May C, et al. What is the work of being a patient when using communication technology to access healthcare in an orthopaedic setting? A systematic review and meta-synthesis of qualitative studies. PROSPERO CRD42018100896 2018 [Available from: http://www.crd.york.ac.uk/PROSPERO/display_record.php?ID=CRD42018100896.
22. Gilbert A, Jones J, Stokes M, et al. Protocol for the CONNECT Project: a mixed methods study investigating patient preferences for communication technology use in orthopaedic rehabilitation consultations. *BMJ Open* 2019;(Epub ahead of Print) doi: 10.1136/bmjopen-2019-035210
23. CASP. CASP Qualitative Checklist [Available from: https://casp-uk.net/wp-content/uploads/2018/01/CASP-Qualitative-Checklist-2018.pdf accessed 18th June 2019.
24. Harrison R, Macfarlane A, Murray E, et al. Patients' perceptions of joint teleconsultations: a qualitative evaluation. *Health Expectations: An International Journal Of Public Participation In Health Care And Health Policy* 2006;9(1):81-90. doi: 10.1111/j.1369-7625.2006.00368.x
25. Wallace P, Haines A, Harrison R, et al. Joint teleconsultations (virtual outreach) versus standard outpatient appointments for patients referred by their general practitioner for a specialist opinion: a randomised trial. *The Lancet* 2002;359(9322):1961-68. doi: 10.1016/S0140-6736(02)08828-1
26. Young L, Siden H, Tredwell S. Post-surgical telehealth support for children and family care-givers. *Journal of Telemedicine and Telecare* 2007;13(1):15-19. doi: 10.1258/135763307779701149
27. Eriksson L, Lindström B, Ekenberg L. Patients' experiences of telerehabilitation at home after shoulder joint replacement. *Journal Of Telemedicine And Telecare* 2011;17(1):25-30. doi: 10.1258/jtt.2010.100317
28. Cranen K, Drossaert CHC, Brinkman ES, et al. An exploration of chronic pain patients' perceptions of home telerehabilitation services. *Health Expectations* 2012;15(4):339-50. doi: 10.1111/j.1369-7625.2011.00668.x
29. Kairy D, Tousignant M, Leclerc N, et al. The patient's perspective of in-home telerehabilitation physiotherapy services following total knee arthroplasty. *International Journal of Environmental Research and Public Health* 2013;10(9):3998-4011. doi: 10.3390/ijerph10093998
30. Moffett H, Tousignant M, Nadeau S, et al. In-home telerehabilitation compared with face-to-face rehabilitation after total knee arthroplasty. *J Bone Joint Surg Am* 2015;97(14):1129-41.
31. Pearson J, Richardson J, Calnan M, et al. The acceptability to patients of PhysioDirect telephone assessment and advice services; a qualitative interview study. *BMC Health Services Research* 2016;16:104-04. doi: 10.1186/s12913-016-1349-y
32. Salisbury C, Foster NE, Hopper C, et al. A pragmatic randomised controlled trial of the effectiveness and cost-effectiveness of 'PhysioDirect' telephone assessment and advice services for physiotherapy. *Health Technology Assessment (Winchester, England)* 2013;17(2):1. doi: 10.3310/hta17020
33. Dobson F, Hinman RS, French S, et al. Internet-mediated physiotherapy and pain coping skills training for people with persistent knee pain (IMPACT - Knee pain): A randomised controlled trial protocol. *BMC Musculoskeletal Disorders* 2014;15(1) doi: 10.1186/1471-2474-15-279

For peer review only

34. Lawford BJ, Bennell KL, Hinman RS, et al. “I was really sceptical...But it worked really well”: a qualitative study of patient perceptions of telephone-delivered exercise therapy by physiotherapists for people with knee osteoarthritis. *Osteoarthritis and Cartilage* 2018;26(6):741-50. doi: 10.1016/j.joca.2018.02.909
35. Hinman RS, Lawford BJ, Campbell PK, et al. Telephone-delivered exercise advice and behavior change support by physical therapists for people with knee osteoarthritis: Protocol for the telecare randomized controlled trial. *Physical Therapy* 2017;97(5):524-36. doi: 10.1093/ptj/pzx021
36. Roberts LC, Whittle CT, Cleland J, et al. Measuring Verbal Communication in Initial Physical Therapy Encounters. *Physical Therapy* 2013;93(4):479-91. doi: 10.2522/ptj.20120089
37. Roberts L, Bucksey SJ. Communicating with patients: what happens in practice? [corrected] [published erratum appears in PHYS THER 2007 Jul;87(7):957]. *Physical Therapy* 2007;87(5):586-94.
38. Katherine JH, Carl RM. Managing expectations: cognitive authority and experienced control in complex healthcare processes. *BMC Health Services Research* 2017(1):1. doi: 10.1186/s12913-017-2366-1
39. Ayo N. Understanding health promotion in a neoliberal climate and the making of health conscious citizens, 2012:99-105.
40. Ellis J, Boger E, Latter S, et al. Conceptualisation of the ‘good’ self-manager: A qualitative investigation of stakeholder views on the self-management of long-term health conditions. *Social Science & Medicine* 2017;176:25-33. doi: 10.1016/j.socscimed.2017.01.018
41. Kaambwa B, Ratcliffe J, Shulver W, et al. Investigating the preferences of older people for telehealth as a new model of health care service delivery: A discrete choice experiment. *Journal of Telemedicine and Telecare* 2016;23(2):301-13. doi: 10.1177/1357633X16637725
42. Andreassen HK, Dyb K, Pope CJ, et al. Digitized patient–provider interaction: How does it matter? A qualitative meta-synthesis. *Social Science and Medicine* 2018;215:36-44. doi: 10.1016/j.socscimed.2018.08.036
43. Greenhalgh T, Wherton J, Papoutsi C, et al. Beyond adoption: A new framework for theorizing and evaluating nonadoption, abandonment, and challenges to the scale-up, spread, and sustainability of health and care technologies. *Journal of Medical Internet Research* 2017;19(11) doi: 10.2196/jmir.8775
44. Sav A, McMillan SS, Hunter B, et al. Burden of treatment for chronic illness: A concept analysis and review of the literature. *Health Expectations* 2015;18(3):312-24. doi: 10.1111/hex.12046
45. Gibbons CJ, Kenning C, Coventry PA, et al. Development of a Multimorbidity Illness Perceptions Scale (MULTIPleS). *Plos One* 2013;8(12)e81852 doi: 10.1371/journal.pone.0081852
46. Boyd CM, Wolff JL, Weiss C, et al. Healthcare task difficulty among older adults with multimorbidity. *Medical Care* 2014;52(3 SUPPL. 2):S118-S25. doi: 10.1097/MLR.0b013e3182a977da
47. Tran VT, Barnes C, Ravaud P, et al. Adaptation and validation of the Treatment Burden Questionnaire (TBQ) in English using an internet platform. *BMC Medicine* 2014;12(1) doi: 10.1186/1741-7015-12-109
48. Eton DT, Yost KJ, Lai J-s, et al. Development and validation of the Patient Experience with Treatment and Self-management (PETS): a patient-reported measure of treatment burden, 2017:489-503.
49. Duncan P, Murphy M, Man MS, et al. Development and validation of the Multimorbidity Treatment Burden Questionnaire (MTBQ). *BMJ Open* 2018;8(4) doi: 10.1136/bmjopen-2017-019413

For peer review only

PRISMA 2009 Checklist

Section/topic	#	Checklist item	Reported on page #
**TITLE**			
Title	1	Identify the report as a systematic review, meta-analysis, or both.	1
**ABSTRACT**			
Structured summary	2	Provide a structured summary including, as applicable: background; objectives; data sources; study eligibility criteria, participants, and interventions; study appraisal and synthesis methods; results; limitations; conclusions and implications of key findings; systematic review registration number.	1
**INTRODUCTION**			
Rationale	3	Describe the rationale for the review in the context of what is already known.	3-4
Objectives	4	Provide an explicit statement of questions being addressed with reference to participants, interventions, comparisons, outcomes, and study design (PICOS).	5
**METHODS**			
Protocol and registration	5	Indicate if a review protocol exists, if and where it can be accessed (e.g., Web address), and, if available, provide registration information including registration number.	4
Eligibility criteria	6	Specify study characteristics (e.g., PICOS, length of follow-up) and report characteristics (e.g., years considered, language, publication status) used as criteria for eligibility, giving rationale.	5
Information sources	7	Describe all information sources (e.g., databases with dates of coverage, contact with study authors to identify additional studies) in the search and date last searched.	4
Search	8	Present full electronic search strategy for at least one database, including any limits used, such that it could be repeated.	Supplementary material 1
Study selection	9	State the process for selecting studies (i.e., screening, eligibility, included in systematic review, and, if applicable, included in the meta-analysis).	4
Data collection process	10	Describe method of data extraction from reports (e.g., piloted forms, independently, in duplicate) and any processes for obtaining and confirming data from investigators.	5
Data items	11	List and define all variables for which data were sought (e.g., PICOS, funding sources) and any assumptions and simplifications made.	5
Risk of bias in individual studies	12	Describe methods used for assessing risk of bias of individual studies (including specification of whether this was done at the study or outcome level), and how this information is to be used in any data synthesis.	4 & 9
Summary measures	13	State the principal summary measures (e.g., risk ratio, difference in means).	9
Synthesis of results	14	Describe the methods of handling data and combining results of studies, if done, including measures of consistency (e.g., I^2) for each meta-analysis.	9

For peer review only - http://bmjopen.bmj.com/site/about/guidelines.xhtml

PRISMA 2009 Checklist

Section/topic	#	Checklist item	Reported on page #
Risk of bias across studies	15	Specify any assessment of risk of bias that may affect the cumulative evidence (e.g., publication bias, selective reporting within studies).	9
Additional analyses	16	Describe methods of additional analyses (e.g., sensitivity or subgroup analyses, meta-regression), if done, indicating which were pre-specified.	9
**RESULTS**			
Study selection	17	Give numbers of studies screened, assessed for eligibility, and included in the review, with reasons for exclusions at each stage, ideally with a flow diagram.	6
Study characteristics	18	For each study, present characteristics for which data were extracted (e.g., study size, PICOS, follow-up period) and provide the citations.	7-8
Risk of bias within studies	19	Present data on risk of bias of each study and, if available, any outcome level assessment (see item 12).	9
Results of individual studies	20	For all outcomes considered (benefits or harms), present, for each study: (a) simple summary data for each intervention group (b) effect estimates and confidence intervals, ideally with a forest plot.	n/a
Synthesis of results	21	Present results of each meta-analysis done, including confidence intervals and measures of consistency.	n/a
Risk of bias across studies	22	Present results of any assessment of risk of bias across studies (see Item 15).	9
Additional analysis	23	Give results of additional analyses, if done (e.g., sensitivity or subgroup analyses, meta-regression [see Item 16]).	n/a
**DISCUSSION**			
Summary of evidence	24	Summarize the main findings including the strength of evidence for each main outcome; consider their relevance to key groups (e.g., healthcare providers, users, and policy makers).	13-16
Limitations	25	Discuss limitations at study and outcome level (e.g., risk of bias), and at review-level (e.g., incomplete retrieval of identified research, reporting bias).	16
Conclusions	26	Provide a general interpretation of the results in the context of other evidence, and implications for future research.	16-17
**FUNDING**			
Funding	27	Describe sources of funding for the systematic review and other support (e.g., supply of data); role of funders for the systematic review.	17

From: Moher D, Liberati A, Tetzlaff J, Altman DG, The PRISMA Group (2009). Preferred Reporting Items for Systematic Reviews and Meta-Analyses: The PRISMA Statement. PLoS Med 6(7): e1000097. doi:10.1371/journal.pmed1000097

For more information, visit: www.prisma-statement.org.

For peer review only - http://bmjopen.bmj.com/site/about/guidelines.xhtml

The CONNECT Project Phase 1 Search Strategy

Searches

English Language studies will be searched for with no date restrictions. The following databases will be searched using a combination of keywords and database specific subject headings: MEDLINE, AMED, CINAHL, PsycINFO and Scopus. Forward and backwards reference screening of identified papers and relevant systematic reviews will be completed using Web of Science.

Types of study to be included

Qualitative studies or studies with a qualitative component (use of interviews or focus groups)

Condition or domain being studied

Orthopaedics and musculoskeletal pathologies

Participants/population

Inclusion:
• Full text academic papers.
• Patients with an orthopaedic / musculosketal problem
• Studies reporting patients accessing clinical care (assessment / rehabilitation) through the use of real time communication technology (eg telephone, videoconferencing) in an orthopaedic / musculoskeletal setting.
• Qualitative studies or studies with a qualitative component (use of interviews or focus groups)

Exclusion:
• Conference abstracts
• Participants without an orthopaedic / musculoskeletal complaint
• Quantitative studies
• Studies not reporting patient viewpoints

Intervention(s), exposure(s)

Inclusion: Participants accessing clinical care (assessment / rehabilitation) through the use of communication technology in real time (eg telephone, videoconferencing) in an orthopaedic / musculoskeletal setting.

Comparator(s)/control

N/A

Context

Studies will be included providing they report the perspectives of patients regarding access to musculoskeletal / orthopaedic assessment / rehabilitation. Studies may also contain perspectives of clinicians in addition to the viewpoints of patients.

Main outcome(s)

Patient experience and workload of using communication technology to access musculoskeletal / orthopaedic assessment / rehabilitation.

Timing and effect measures

Not applicable.

Additional outcome(s)

None

Timing and effect measures

None

Data extraction (selection and coding)

Two researchers will independently identify full text papers meeting the inclusion criteria. Discrepancies arising regarding selection will be resolved through discussion with a third researcher.

Risk of bias (quality) assessment

Two researchers will independently screen the full text included studies for risk of bias. The Critical Appraisal Skills Programme Qualitative Tool will be used. Discrepancies arising regarding risk of bias will be resolved through discussion with a third researcher.

Strategy for data synthesis

All studies included for full text review will be analysed. Data extracted from the Methods, Results, Discussion and Conclusion sections will be analysed by using an attribution analysis. A taxonomy of attributions will be thematically analysed using NVIVO software in accordance with Normalisation Process Theory.

Search Terms

Database	Search Term
MEDLINE	(communication technology OR e-health OR telecare OR telemedicine OR telehealth OR telemonitoring OR videoconferencing OR real time videoconferencing OR real time 1:1 videoconferencing OR telecommunication OR virtual OR (MH "telemedicine") OR (MH "remote consultation") OR (MH "videoconferencing")) AND (Focus group OR Focus groups OR observation OR Ethnography OR Ethnographic OR Phenomenology OR Phenomenological OR Lived experience OR Grounded theory OR Thematic analysis OR Conversation analysis

	OR Framework analysis OR (MH "Qualitative Research")) AND (physio OR physiotherapy OR physical therapy OR rehab OR rehabilitation)
AMED	(communication technology OR e-health OR telecare OR telemedicine OR telehealth OR telemonitoring OR videoconferencing OR real time videoconferencing OR real time 1:1 videoconferencing OR telecommunication OR virtual OR (SU (telemedicine) OR (SU (technology medical))) AND (Focus group OR Focus groups OR observation OR Ethnography OR Ethnographic OR Phenomenology OR Phenomenological OR Lived experience OR Grounded theory OR Thematic analysis OR Conversation analysis OR Framework analysis) AND (physio OR physiotherapy OR physical therapy OR rehab OR rehabilitation)
CINAHL	(communication technology OR e-health OR telecare OR telemedicine OR telehealth OR telemonitoring OR videoconferencing OR real time videoconferencing OR real time 1:1 videoconferencing OR telecommunication OR virtual OR (MH “Videoconferencing”) OR (MH “Remote Consultation”)) AND (Focus group OR Focus groups OR observation OR Ethnography OR Ethnographic OR Phenomenology OR Phenomenological OR Lived experience OR Grounded theory OR Thematic analysis OR Conversation analysis OR Framework analysis OR (MH “qualitative studies”)) AND (physio OR physiotherapy OR physical therapy OR rehab OR rehabilitation)
PsychINFO	(communication technology OR e-health OR telecare OR telemedicine OR telehealth OR telemonitoring OR videoconferencing OR real time videoconferencing OR real time 1:1 videoconferencing OR telecommunication OR virtual OR (DE "Telemedicine")) AND (Focus group OR Focus groups OR observation OR Ethnography OR Ethnographic OR Phenomenology OR Phenomenological OR Lived experience OR Grounded theory OR Thematic analysis OR Conversation analysis OR Framework analysis OR DE "Qualitative Research")) AND (physio OR physiotherapy OR physical therapy OR rehab OR rehabilitation)

SCOPUS	1. (TITLE-ABS-KEY (“communication technology” OR e-health OR telecare OR telemedicine OR telehealth OR telemonitoring OR videoconferencing OR “real time videoconferencing” OR “real time 1:1 videoconferencing” OR telecommunication OR virtual))<br>2. (TITLE-ABS-KEY (“Focus group” OR “Focus groups” OR observation OR Ethnography OR Ethnographic OR Phenomenology OR Phenomenological OR “Lived experience” OR “Grounded theory” OR “Thematic analysis” OR “Conversation analysis” OR “Framework analysis”))<br>3. (TITLE-ABS-KEY (physio OR physiotherapy OR physical therapy OR rehab OR rehabilitation)<br>4. #1 AND #2 AND #3

For peer review only

BMJ Open

The use of virtual consultations for orthopaedics rehabilitation consultations: How do changes in the work of being a patient influence patient preferences? A systematic review and qualitative synthesis

Journal:	*BMJ Open*
Manuscript ID	bmjopen-2019-036197.R1
Article Type:	Original research
Date Submitted by the Author:	18-May-2020
Complete List of Authors:	Gilbert, Anthony; Royal National Orthopaedic Hospital Stanmore, Therapies Department; University of Southampton, Faculty of Health Sciences<br>Jones, Jeremy; University of Southampton, Faculty of Health Sciences<br>Jaggi, Anju; Royal National Orthopaedic Hospital Stanmore, Therapies Department<br>May, Carl; London School of Hygiene and Tropical Medicine Faculty of Epidemiology and Population Health
**Primary Subject Heading**:	Qualitative research
Secondary Subject Heading:	Health services research
Keywords:	Telemedicine < BIOTECHNOLOGY & BIOINFORMATICS, ORTHOPAEDIC & TRAUMA SURGERY, QUALITATIVE RESEARCH, REHABILITATION MEDICINE

SCHOLARONE™
Manuscripts

BMJ

I, the Submitting Author has the right to grant and does grant on behalf of all authors of the Work (as defined in the below author licence), an exclusive licence and/or a non-exclusive licence for contributions from authors who are: i) UK Crown employees; ii) where BMJ has agreed a CC-BY licence shall apply, and/or iii) in accordance with the terms applicable for US Federal Government officers or employees acting as part of their official duties; on a worldwide, perpetual, irrevocable, royalty-free basis to BMJ Publishing Group Ltd ("BMJ") its licensees and where the relevant Journal is co-owned by BMJ to the co-owners of the Journal, to publish the Work in this journal and any other BMJ products and to exploit all rights, as set out in our licence.

The Submitting Author accepts and understands that any supply made under these terms is made by BMJ to the Submitting Author unless you are acting as an employee on behalf of your employer or a postgraduate student of an affiliated institution which is paying any applicable article publishing charge ("APC") for Open Access articles. Where the Submitting Author wishes to make the Work available on an Open Access basis (and intends to pay the relevant APC), the terms of reuse of such Open Access shall be governed by a Creative Commons licence – details of these licences and which Creative Commons licence will apply to this Work are set out in our licence referred to above.

Other than as permitted in any relevant BMJ Author's Self Archiving Policies, I confirm this Work has not been accepted for publication elsewhere, is not being considered for publication elsewhere and does not duplicate material already published. I confirm all authors consent to publication of this Work and authorise the granting of this licence.

For peer review only

The use of virtual consultations for orthopaedics rehabilitation consultations: How do changes in the work of being a patient influence patient preferences? A systematic review and qualitative synthesis

Authors

1. Anthony Gilbert[1, 2]
2. Jeremy Jones[2]
3. Anju Jaggi[1]
4. Carl R May[3]

Author Affiliations

1. Therapies Department, Royal National Orthopaedic Hospital, Stanmore, UK

2. School of Health Sciences, Faculty of Environment and Life Sciences, University of Southampton, Southampton, UK

3. Faculty of Public Health and Policy, London School of Hygiene and Tropical Medicine, London, UK

Corresponding author: Anthony Gilbert anthony.gilbert@nhs.net

For peer review only

Abstract

Objectives

To systematically review qualitative studies reporting the use of virtual consultations within an orthopaedic rehabilitation setting and to understand how it's use changes the work required of patients.

Methods

Following the Preferred Reporting Items for Systematic Reviews and Meta-Analysis Statement we conducted a systematic review of papers to answer the research question 'How do changes in the work of being a patient when using communication technology influence patient preferences?' Electronic databases were searched for studies meeting the inclusion criteria in April 2020.

Results

The search strategy identified 2057 research articles from the database search. A review of titles and abstracts using the inclusion criteria yielded 21 articles for full text review. Nine studies were included in the final analysis. Six studies explored real time videoconferencing and three explored telephone consultations. The use of communication technology changes the work required of patients. Such changes will impact on expectations for care, resources required of patients, the environment of receiving care and patient-clinician interactions. This adjustment of the work required of patients who access orthopaedic rehabilitation using communication technology will

impact on their experience of receiving care. It is proposed that changes in the work of being a patient will influence preferences for or against the use of communication technology consultations for orthopaedic rehabilitation.

Conclusion

We found that the use of communication technology changes the work of being a patient. The change in work required of patients can be both burdensome (it makes it harder for patients to access their care) and beneficial (it makes it easier for patients to access their care). This change will likely to influence preferences. Keeping the concept of patient work at the heart of pathway redesign is likely to be a key consideration to ensure successful implementation.

Registration

PROSPERO registration number: ID=CRD42018100896.

Strength and limitations of this study

- A taxonomy of patient work will assist in understanding implementation processes.
- The use of middle range theory has been employed to guide theorization of the data.
- A secondary analysis of data has been employed to explain concepts which the authors had not originally intended.
- The date range of included studies (2005-2019) include a range of technologies including the use of bespoke software which may present different challenges to modern off the shelf software.

For peer review only

Introduction

<u>Background</u>

The NHS Long Term Plan[1], The United Kingdom's health service's plan to 'make the NHS fit for the future of patients', advocates digital-first primary care and envisions the use of e-consultations to become a new option for every patient. The outbreak of 2019 novel coronavirus (COVID 19) was first reported in Wuhan, China and reached the United Kingdom on the 31st January 2020. The COVID-19 virus spreads primarily through droplets of saliva or discharge from the nose when an infected person coughs or sneezes. Social distancing measures have been established with the UK public being placed on 'lockdown' from the 23rd March 2020[3] to avoid transmission of the disease. Healthcare organisations have subsequently embraced the use of virtual consultations to comply with these social distancing measures[4].Virtual consultations can support the management of patients with long term conditions such as musculoskeletal disease[5] where long term management may require repeat visits for appointments with healthcare practitioners. Virtual consultations can support communication from a distance and has been demonstrated as a useful digital innovation that has enabled patients to attend appointments during the COVID-19 pandemic[4].

There are examples of virtual consultations in practice. The PhysioDirect telephone and advice service[7] is an example that was found to be safe and resulted in equivalent outcomes to face to face appointments for patients with musculoskeletal disorders. The visual component offered with videoconferencing software offers distinct advantages over telephone consultations[8]. Research has been conducted investigating patients using Skype, a free to access videoconferencing software, to access care[9]. Patients who received telerehabilitation for knee arthritis via SKYPE[10] found it to be feasible and acceptable. The Virtual Online Consultations-Advantages and Limitations (VOCAL) study[11] found video outpatient consultations to be safe, effective and convenient in appropriate situations.

The process of implementing a new intervention (such as the introduction of virtual consultations in healthcare) has been demonstrated to be dependent on how the intervention is operationalised by its users[12], the work people do when they implement a new intervention[13] and the mobilisation of resources over time[14] across different settings[15]. Normalisation Process Theory (NPT) frames implementation processes through its focus on the things people *do* when they implement a new intervention in practice. One study investigated nurse call takers conducting a physical assessment of patients' over a telephone helpline[16]. The study reported nurses' interactions with patients as they instructed them over the phone to perform physical manipulations. The accomplishment of a physical examination required work from patients that differs to face to face consultations. Burden of treatment theory[17] explains how the capacity for action interacts with the work that stems from healthcare. Burden of treatment has been demonstrated to arise when the workload demands exceeds the capacity for patients with COPD and lung cancer[18]. An understanding of the factors that contribute to a change in the work for patients using virtual consultations is an important consideration for patient experience

Research conducted in the UK found that the majority of people say they would use video consultations to consult their General Practitioner about minor ailments and ongoing condition[19]. A proportion (approximately 35%) would not use this modality. Our previous research investigated whether patients preferred face to face or virtual consultations[20]: patients with atraumatic shoulder instability were offered the choice between SKYPE and face to face follow up rehabilitation appointments. Half of patients preferred to see their rehabilitation professional in person[20] in part

For peer review only

due to not having access and knowing how to use the software and equipment. The use of SKYPE changed what patients needed to do to engage in their care in our small study and this influenced their choice on whether or not to use it.

Preferences are a set of complex factors that may include enjoyment comparisons *(x to y is preferred if someone enjoys x more than they enjoy y)*, comparative evaluations *(x to y is preferred if someone thinks x is better than y)*, favouring *(selecting x over y because x has a particular set of characteristics)* or choice ranking *(x is chosen over y if and only if they are faced with a choice of x over y)*[21]. To get past the complexities of preferences, preferences can be defined as a 'total subjective comparative evaluation'[22]. In essence, someone will prefer x over y after consideration of the alternatives, the actions, the state of affairs and the consequences of choosing each alternative. In this paper we are interested in understanding how patient work influences patient preferences.

Aims of this review

This paper reviews qualitative literature on the use of communication technology for patients in an orthopaedic rehabilitation setting to understand how the work of being a patient influences preference. The purpose of this paper is to develop a taxonomy of tasks required of patients using communication technology. We then consider how factors relating to these tasks influence the comparative evaluation patients face when offered the choice of a communication technology or a face to face consultation for orthopaedic rehabilitation.

Methods

A systematic review was conducted using the PRISMA approach in order to answer the research question: *How do changes in the work of being a patient when using virtual consultations influence patient preferences?* This review was registered on the International prospective register of systematic reviews (PROSPERO registration number: ID=CRD42018100896)[23]. The protocol for this review, which forms phase 1 of the CONNECT Project, has previously been published[24].

MEDLINE, AMED, CINAHL, PsychINFO and SCOPUS were searched from inception on the 4th April 2020. Full search terms and the search strategy is available to view in Supplementary Material (See Supplementary Material 1). Articles were screened independently by two authors (AWG and AJ) with a third author (JJ) available to discuss any discrepancies (See Figure 1 in Supplementary Material 2 for reporting).

Studies were eligible for inclusion providing they met the criteria for inclusion shown in Table 1. Relevant studies were firstly screened by their title and then by their abstract. Remaining texts were then read in full with all texts retained after this point for qualitative synthesis. Risk of bias was screened using the CASP tool for qualitative studies[25]. A discussion was held, between two authors (AWG and AJ) with a third author (JJ) available to discuss any discrepancies, to decide whether included studies were of sufficient quality to include in the review.

For peer review only

Table 1: Eligibility Criteria of Studies

Inclusion:	Exclusion:
• Full text English Language academic papers from inception to 6th April 2020.<br>• Patients with an orthopaedic / musculoskeletal problem<br>• Studies reporting patients accessing physical assessment / rehabilitation through the use of virtual consultations (e.g. telephone, videoconferencing) in an orthopaedic / musculoskeletal setting.<br>• Qualitative studies or studies with a qualitative component that focuses on the patient viewpoint of accessing virtual consultations.	• Conference abstracts<br>• Participants without an orthopaedic / musculoskeletal complaint<br>• Quantitative studies<br>• Studies not reporting patient viewpoints

Full texts were uploaded to QSR NVIVO Software (QSR International Pty Ltd. Version 12, 2018). NVIVO was used to collect and organize data from the results, discussion and conclusion sections of each paper. Each sentence from the included sections were coded on a line by line basis. The codes were labelled using a description of the content of the respective sentence. Data analysis subsequently took three forms: firstly, two authors (AWG and CRM) conducted a thematic analysis of codes. This was undertaken to familiarize the authors with the content of the papers. For the second iteration of coding the following was considered: *what is the work of being a patient when using virtual consultations?* Codes were then organized into groups depicting the *type* of work required of patients when using virtual consultations to access healthcare. The two authors (AWG and CRM) then considered the question: *how might the work of being a patient when using virtual consultations influence patient preference?* The data were revisited and theoretical ideas arising from the data were discussed between AWG & CRM. From here themes, empirical regularities in the data, were identified and characterised. Finally, themes arising from the data were mapped out in the form of a model to demonstrate how, based on the included papers, the change in the work of being a patient might influence preference for virtual consultations.

For peer review only

Results

Study Selection

Systematic search identified 1,655 references (after de-duplication) of which 1,634 were excluded on the basis of titles and abstracts and a further 12 excluded at full text review. As a result 9 papers were included in the review. Of the eight papers, two originated from Australia[10 36], 2 from Canada[28 31] and 3 two from England[20 26 33] and with one from Sweden[29] and one from the Netherlands[30]. Six studies explored real time videoconferencing[10 20 26 28-31] and three explored telephone consultations[28 33 36]. Study demographics are shown in Table 2. All studies were screened using the CASP tool for qualitative studies[27] and all were deemed by the authors to be of sufficient quality and therefore retained for analysis.

For peer review only

Table 2 – Study Characteristics.

Included Study	Study Setting	Study Purpose	Technology Used	Participants
Harrison et al 2006[26]	Joint teleconsultations between the patient and their GP and a hospital specialist (England)	To explore patients' experiences of joint teleconferenced consultations	ISDN2 link and off-the-shelf videoconferencing software	28 patients who were enrolled in the Virtual Outreach Randomized Trial[27]. Six patients had a generic orthopaedic diagnosis.
Young et al 2007[28]	Telephone and videophone follow up after scoliosis surgery (Canada)	To better understand the relative effectiveness of two types of telehealth technology, telephone versus videophone, following a child's scoliosis surgery from the perspective of patients and care-givers	For the videophone group patients were provided with a videophone (KXC-AP150, Panasonic, Japan). For the telephone group patients used an ordinary telephone line	43 patients and their families (dyads) who had undergone scoliosis correction surgery. 21 dyads received videophone support and 22 dyads who received telephone support.
Eriksson et al 2011[29]	Video-based physiotherapy at the patient's home for two months after a shoulder replacement (Sweden)	To describe patients' experiences of physiotherapy at home by video-link after a shoulder replacement	Standard commercial videoconferencing units (e.g. Tandberg 800, Sony PCS-50, Polycom VSX 3000)	10 Adults who had undergone a shoulder replacement.
Cranen et al 2011[30]	Telerehabilitation services at a rehabilitation centre (Netherlands)	To explore patients perceptions regarding prospective rehabilitation services and the factors that facilitate or impede patients' intentions to use these services	Home based treatment by means of (unspecified) web cam treatments	25 chronic pain patients from a rehabilitation centre.
Kairy et al 2013[31]	Telerehabilitation between the patient at home and the physical therapist at the hospital (Canada)	To better understand the patient's experience of home telerehabilitation	Internet access and the telerehabilitation platform was installed in the patient's home as reported in[29].	5 patients who had previously received in-home telerehabilitation post knee arthroplasty. Patients were

For peer review only

			The telerehabilitation device was custom built for the study	selected from a pool of participants from the experimental arm of a RCT for in-home telerehabilitation[32].
Pearson et al 2016[33]	Telephone based physiotherapy between a patient and a senior physiotherapist (England)	To describe key variables that determined patient acceptability of the PhysioDirect service and to understand how the patient experience differed from those accessing usual physiotherapy care.	Telephone	57 patients with a musculoskeletal problem. Participants were recruited from the PhysioDirect study[34].
Hinman et al 2017[10]	SKYPE mediated physiotherapy consultations between the patient at home and the physiotherapist (Australia)	To explore the experience of patients and physical therapists with Skype for exercise management of knee osteoarthritis (OA)	SKYPE software	12 patients with a diagnosis of knee osteoarthritis. Participants were key informants from an RCT[35].
Lawford et al 2018[36]	Exercise therapy for people with knee arthritis via telephone (Australia)	To explore people's perceptions of exercise therapy delivered by physiotherapists via telephone.	Telephone	20 patients with knee osteoarthritis. Participants with knee OA were recruited as key informants from an RCT[37].

For peer review only

Gilbert et al 2019[20]	Follow up consultations for patients after a period of inpatient rehabilitation for atraumatic shoulder instability.	To explore reasons behind acceptability of SKYPE follow up consultations	SKYPE software	7 patients chose a SKYPE consultation, 6 patients chose a face to face consultation. In addition, 8 clinicians were interviewed.

For peer review only

Worked Example of Data Analysis

Data from the nine studies were synthesized. All data were treated to the same three step process. An exemplar is demonstrated below using data from Eriksson et al[29]:

1. Data identified (initial line by line identification)

Inability to touch the patient meant therapists were forced to rely more on their subjective assessment of the patient, leading them to spend more time talking with and listening to patients.

2. Data characterized (initial line by line coding)

Code assigned: *Therapists were unable to use 'hands on' during assessment.*

3. Data theorized (consideration of the question: *what is the work of being a patient when using virtual consultations?*)

Patients have to present themselves in a different way during assessment via VC.

Data from the papers are presented in Table 3.

For peer review only

Table 3 - Factors that may affect patient preference for virtual consultations and considerations for virtual consultations

Finding	Construct	Results from included papers: factors that contribute towards the work of being a patient when using communication technology	Considerations for virtual consultations
Preferences are shaped by the requirements of the consultation how these change the work	The processes that change	Patients were able to engage in consultation from different places[10]. Using virtual consultations required patients to arrange for additional equipment in the home[31]. They were required to log in to an account[30] and to learn how to use the communication technology[10].	• Consider the impact of changing processes on patients.<br>• Offer troubleshooting for logging in and how to use the equipment.<br>• Consider offering guidance surrounding the suitability of different locations when engaging in virtual consultations.
	The skills & expertise that is required	As patients moved away from physically facilitated exercises there was the requirement to adjust[29], overcome patient-clinician communication difficulties over video-call[26 28] and phone call[33] and face an increased reliance on them to communicate information[10]. In the absence of hands on treatment more emphasis is placed on patients completing exercises[10]. Patients need to self-assess when they cannot be physically assessed by a therapist[10]. Patients may need to adapt to clinicians who do not have adequate communication skills or training for using virtual consultations[36]. Patients may be encouraged to self-monitor improvements more than if they were seen face to face[33].	• Brief and support patients on the changes in style of communication.<br>• Facilitate patients to communicate their problems through a virtual consultation.<br>• Facilitate self-assessment of patients in the absence of clinician's 'hands-on' care.<br>• Facilitate and provide guidance on self-assessment and ongoing monitoring.<br>• Design personalized exercise regimens that are suitable for the patient's clinical problem and their home environment
Preferences are shaped by the resources that are required of patients	Logistics	Use of virtual consultations helps to avoid of transportation issues[10 20 29-31 36], reduces travel times[30] for both patients and carers and can increase access to services[10].	• Consider offering virtual consultations for patients who experience difficulty with travel

For peer review only

	Time	The ease in which exercises can be integrated into home routine[20 30] and through avoidance of travel provides additional time and energy for other activities[29]. Patients valued being able to wait for their appointment in their own chosen environment rather than in the clinic[10 26 33].	• Consider conflicting demands for patients.<br>• Consider the impact of travel and time on patient symptoms.<br>• Consider the impact of patient comfort when waiting for their appointment.
Preferences are shaped by the work required due to the changes in the environment	Setting for physical rehabilitation	Patients had to find ways to overcome a lack of space[10 30] and equipment[10 31] at home. Patients were required to integrate their rehabilitation in the home environment[10 36].	• Support patients to establish a suitable rehabilitation environment at home.<br>• Design treatment regimens based on the patients access to rehabilitation equipment.<br>• Support patients to integrate rehabilitation within the home environment.
	Setting for virtual consultation	At times the rehab was impaired due to technical difficulties[10] and patients felt they missed learning through fellow sufferer contact through not attending the clinic and would need to seek this elsewhere[30].	• Offer troubleshooting when faced with technical difficulties<br>• Consider offering peer support groups for patients who are unable to physically attend the clinic.
	Hardware and software	Patients needed to be supported to access[10 31] and use the equipment[10 20 28 30] and manage to real-time troubleshoot connection problems as they arose[10 28 29 31].	• Consider offering equipment based on the patient's needs.<br>• Tailor support for equipment use based on patient's skill set.<br>• Offer troubleshooting when faced with technical difficulties.
Preferences are shaped by the work that goes into maintaining adequate interactions	Interactions	Patients may have to focus additional attention when communicating over a stutter connection[10 29] or when faced with a language barrier[28]. Patients may need to rely on additional non-verbal communication when communicating over a screen[26]. Patients who feel alienated[30] or detached[30 33] or expect	• Clearly communicate when the connection is impaired; be prepared to abandon and reboot the virtual consultation as required.<br>• Be prepared to emphasize the use of non-verbal communication.

For peer review only

		hands on care[10 28 33] may need to invest additional effort in developing an effective therapeutic the patient clinician relationship.	• Have an awareness of patient preferences; patients who prefer face to face care may require additional input to develop a therapeutic relationship.

For peer review only

Synthesis of Results

Theme 1: Requirements of rehabilitation

1.1 The processes that change

The use of virtual consultations within the treatment pathway required additional steps for patients, such as logging in[30] and setting the software up[10]. Some patients valued the portability of using Skype[10] and found that they could use it across different settings[20] to fulfil the purposes of the consultation. Patients valued the opportunity to run through the processes of using SKYPE for the first time in the form of a 'dummy run'[20].

1.2 The skills & expertise that is required

The use of virtual consultations changed the skills patients needed. Video communication required specific communication skills that included listening with close attention with no interruptions[29]. The gaze of the patients and clinicians were used to signal the start and end of conversations[29]. Patients and their families found it challenging to express how they felt from a distance and were reliant on the visual capabilities of the technology[28]. The lack of visual information was a concern for patients in the PhysioDirect service[33] who did not have visual cues and physical contact. The lack of physical contact meant that therapists were more reliant on information shared by patients rather than those derived from physical tasks[10]. Therapist focussed on more effortful treatments such as exercises and self-management rather than providing them with hands on care[10]Traditional face-to-face interaction is well established and accepted. It was recognised that virtual communication required different skills and therapists' training needs, to ensure effective communication with patients, were considered in one study of telephone consultations[36]. Traditional physiotherapy patient assessment (such as 'hands on' palpation of a joint) is not possible via Skype. As a result of this patients were taught to self-palpate under guidance[10] and instructed how to demonstrate their range of movement over the screen. It is self-evident that visual assessment was not possible over telephone[28 33 36] and this required good communication from both therapists and patients to describe the movements. Patients felt they did not need 'hands on' care when they were seen by an experienced therapist[29] and clinicians were more likely to encourage self-management and exercises when they were seen virtually[33].

Theme 2: Resources

2.1 Logistics

Patients who underwent virtual consultations experienced reduced travel times and transportation issues[10 29-31 36] and was often seen as more convenient for patients, particularly those who suffered from chronic pain[10]. Virtual consultations enabled patients to access health services more easily[26 31]. Problems did arise with the PhysioDirect service where patients were unable to get through requiring them having to make multiple calls to speak to a therapist.

2.2 Time

Virtual consultations offered flexibility[36]: *'If I know I'm stuck at work and I can't get to see someone [the telephone] would be a good option…I can ring someone or have an appointment on the phone, and be at work doing what I need to do, and still have my appointment.'* It was particularly useful for patients who had multiple commitments: *'Because life's so busy in general too, so to be able to speak to*

For peer review only

somebody in your home and then you can go on with your, you know, your next thing, is just wonderful…it just opens another brilliant option for people' as it provided more time for other activities and to integrate rehabilitation into daily life[29].

Theme 3: Environment

3.1 Setting for rehabilitation

Rehabilitation in the home was welcomed by some patients as it gave them the opportunity to rehab within their own environment whereas other patients preferred to keep their home environment separate from the clinical environment[30]. Patients found that they had a lack of space at home compared to the clinic[10 30] and could not access clinic-based equipment[10 31]. Rehabilitation required patients to troubleshoot ways to integrate their rehabilitation tasks within the home[10 36].

3.2 Setting for virtual consultation

Some patients valued fellow sufferer contact and felt that through not physically attending the clinic they missed out on stimuli which kept them motivated. Rehabilitation was impaired when there were issues with connectivity and audio-visual interference disrupted the flow of the consultation[10]. Some patients felt that telerehabilitation was as good as real life and didn't affect the flow of the consultation[29].

3.3 Hardware and Software

Patients who did not have access to equipment for virtual consultation needed to be provided with the required hardware[28 29 31]. In some cases, significant support was required for patients to understand how to use the equipment [30 10 28] and to troubleshoot connection problems when they arose[10 28 29 31]. Overcoming these barriers was an important factor in maintaining the quality of the virtual consultation and is likely to require technical support provided by the clinical team[10].

Theme 4: Interactions

Some patients reported being more relaxed in their own home[10]. One patient, however, felt uncertain about having someone looking into their home and aborted the video consultation[29]. Virtual interactions were impaired at times there was a poor connection[10 29] or a language barrier[28]. These situations demanded additional focus and non-verbal communication[26] from the patient. The therapeutic relationship between patients and clinicians is negatively affected when patients feel alienated[30] or detached[30 33] from their clinician. Patients with an expectation of hands on care[10 20 28 33] found virtual rehabilitation more challenging and may need to invest additional effort to maintain an effective relationship with their therapist.

For peer review only

Discussion

This review synthesized nine qualitative studies reporting the use of virtual consultations in an orthopedic setting. We explored how the use of these technologies impacts on the work of being a patient. All studies in this review demonstrated that adjustments are required of patients to operationalise communication technology for virtually mediated clinical interactions. The adjustments (in the work) that a patient needs to make will have an effect on their experiences of receiving care. These experiences, whether previously lived or anticipated in the future, are likely to influence whether or not an individual finds the use of virtual consultations acceptable. The patient preference for a virtual consultation will depend on individual circumstances. Some of these factors which might influence their decision have been and presented in a conceptual model. The model attempts to demonstrate the relationship between patient work and preference when using communication technology. The model suggests that the use of virtual consultations changes the work of being a patient. The consequences (both positive and negative) of these alterations in work may impact on the patient's experience of receiving their healthcare, their burden of treatment and their ability to engage with their healthcare. This is an important consideration for clinician, managers and policy makers.

Clinicians have to pay more attention to the patient as a result of communicating using technology compared to face to face consultations[9]. This appeared to be at odds with traditional consultations where physiotherapists spoke for half of the allotted time compared to patients who spoke for only 33.1%[38] in initial encounters. A study found, during a follow up session between physiotherapists and patients, that physiotherapists spent twice as much time talking as the patients did and they relied on the use of their hands during the session[39]. In addition to the content within sessions, the relationship experienced between the clinician and the patient may differ during a virtual consultation due some patients being more relaxed at home[9].

Some patients expected 'hands-on' treatment. The transfer of clinician manual therapy towards patient self-palpation[10] and exercise[33] may go against what is expected of therapists. The normative expectations of the patients change as a result of the geographical separation (and physical resources that can be mobilized) between patient and therapist[40]. This places particular emphasis on self-management which shifts the responsibility for health away from the state and onto the individual[41]. This is an important consideration as virtual consultations becomes increasingly used in clinical practice. The additional responsibility of self-management[42], the change in work and tasks required to operationalise communication technology may further burden patients as they are rehabilitated virtually.

Patient viewpoints are important. Kaambwa et al[43] found in their study of older people that patients had strong preference for telehealth services that targeted individuals living in remote regions without easy access to clinic. Our previous research[20] demonstrated that distance to travel to a hospital was not the sole reason leading to the acceptability of Skype consultations and that preference is multi-factorial. We found that having rehabilitation in the patient's own environment was preferred by some although bringing the clinical space into the patient's home can change the meaning of their home for them[44]. Greenhalgh et al[45] considers, amongst other things, *what is expected* of the patient when using new technologies and explains that complex tasks are more likely to lead to non-adoption.

Greenhalgh et al's VOCAL study[46] found that the situations where patients were appropriate for video outpatient consultations only formed a fraction of the overall workload. Such situations

For peer review only

included when close physical examination was not required and when both parties were technically confident and competent. The use of virtual consultations in these situations may increase patient work and therefore contribute towards their burden of treatment. Patients may therefore opt to choose a face to face consultation. Sav et al[47] call for collaborative discussions to help alleviate treatment burden.

Digitally enabled services are a key focus for the UK's National Health Service over the next 10 years[1]. The use of digitally enabled services such as virtual consultations may be useful for some but add to the burden of treatment to others. Tools have been developed to assess burden of treatment[48-52]. Further research investigating the utility of tools such as these may highlight areas where digitally enabled services negatively (or positively) impact on patient experience. The work required and subsequent treatment burden for patients will differ on an individual case by case basis. Table 3 outlines some considerations for clinicians and policymakers considering the use of virtual consultations based on our findings from this systematic review. Further research investigating patient preference will help researchers and clinicians tailor services in a way that suits the need of patients.

Figure 2 in the supplementary material demonstrates how the themes from this review interact with patient preferences. The work required of a patient will influence their expectations of whether or not the use of virtual consultations is acceptable. The logistics and time required of a patient will shape the resources the patient has to dedicate towards their care. The space available and the equipment the patient has access to determines the suitability of the environment. These, coupled with the impact on patient-clinician interactions will determine patient preference for or against virtual consultations. This leads us to our first preposition: Proposition 1: The work required of patients when using virtual consultations will influence their preferences for their use.

Face to face consultations and communication technology consultations have different requirements. Upon choosing a face to face consultation the patient follows the standard pathway. Choosing a communication technology consultation changes what is needed of patients. The change of work demands different skills, processes, expertise, logistical and environmental considerations. This in turn impacts on the nature of the interactions between the patient and their therapist. This leads us to our second preposition: Proposition 2: The preferences regarding the use of virtual consultations will influence the work of being a patient.

The outbreak of COVID-19 has led to a huge upsurge in the interest and importance of virtual consultations in practice[4 53 54]. As such, many more patients have been forced into undergoing virtual consultations than would have otherwise been required. NHSx recently published information governance advice for health and care professionals [55] to facilitate appropriate use of virtual consultations during COVID-19. Future research should carefully evaluate the consequences of rapid virtual consultation implementation to allow for appropriate redesign of services embracing communication technology. Such redesign should consider how the use of these technologies impact on the work of being a patient.

Limitations of this review

Our review is subject to a number of important limitations. We included papers from the UK, Sweden, USA, Canada, the Netherlands and Australia which utilized a variety of communication technologies. The data that underpins our results are a secondary analysis of other previously collected data. We did not have access to the original qualitative datasets, only that presented in the research papers. To arrive at our conclusions, we have subjected the data from the primary studies

For peer review only

to explanatory concepts that the original authors had not intended. The studies spread from 2005 to 2018. During this time technology has advanced considerably and the bespoke software used in the earlier studies (that were developed for the research study) may present different challenges to modern off the shelf software for use with commonly used personal devices such as phones, tablets or computers. It is also important to acknowledge the differences between the different types of technologies. A phone call does not allow for visualization whereas a video call does. Focusing on specific technologies may have generated more applicable results. The original research recruited patients who had opted into these studies. Patients who are satisfied with these technologies are more likely to be recruited to telemedicine studies and may not be a representative sample.

Conclusion

We reviewed eight qualitative studies which reported the use of phone or videocall in orthopaedic care and found that the use of virtual consultations changes the work of being a patient. We identified four different kinds of work relating to: (1) the consultation, (2) the use of resources, (3) changes in the environment and (4) interactions with the healthcare professional. Across all four domains, the change in work required of patients can be both burdensome (it makes it harder for patients to access their care) and beneficial (it makes it easier for patients to access their care). The burden experienced by patients is a result of the relationship between the demands of the work and their capacity to fulfill these demands. Such burden is individual and situational, depending on the clinical requirements and the patient's lifeworld. As a result, we have proposed that the work of being a patient influences their preferences and the resulting choice has consequences on the resulting work that is required of them. Changes in circumstances (such as availability of equipment, understanding of how to use the equipment, requirements of the rehabilitation) may alter what is required both clinically and technologically and influence preferences. This is an important consideration to patients, clinicians, managers and policy makers, especially at a time where the use of technology is being favored during the COVID-19 outbreak. We have demonstrated the importance of considering the work of being a patient when designing and implementing new technologies. Keeping the concept of patient work at the heart of technology implementation is essential to ensure successful uptake in practice.

Patient and Public Involvement

The CONNECT Project Patient and Public Involvement steering group (PPISG) has been set up to provide guidance on the conduct of the research (details available from www.theconnectproject.info). The first meeting of the PPISG was held in August 2016 prior to the submission of the research to the NIHR in May 2017. A discussion was held about the overall research aims which supported the identification of the research questions. The PPISG has supported the design of the overall research plan and will continue to be involved during the development and refinement of each phase prior to the completion of each study protocol. In addition, the PPISG will support the development of the lay-summary outputs to be disseminated to patients and members of the public. Links to research outputs will be made available on the CONNECT Project website available at www.theconnectproject.info.

Acknowledgements

The authors thank Professor Maria Stokes and members of the CONNECT Project Patient and Public Involvement steering group for their invaluable contributions to the overall study design of the CONNECT Project and obtaining funding for the PhD Fellowship. The authors also thank colleagues

For peer review only

within the Therapies Directorate and Research and Innovation Centre at the Royal National Orthopaedic Hospital for their ongoing support.

Contributors

AWG wrote the paper and conceived the project with CRM and JJ. CRM contributed knowledge on systematic reviews and qualitative analysis. AWG and AJ completed the literature search, identification of papers and quality analysis of papers. CRM, JJ, and AJ edited and critically revised the paper. All authors have read and approved the manuscript. AWG is the guarantor of the manuscript.

Funding Statement

Anthony Gilbert is funded by a National Institute for Health Research (NIHR), Clinical Doctoral Research Fellowship for this research project (ICA-CDRF-2017-03-025).

Disclaimer

This paper presents independent research funded by the National Institute for Health Research (NIHR). The views expressed are those of the authors and not necessarily those of the NHS, the NIHR or the Department of Health and Social Care.

Competing Interests

Nil.

Patient Consent for Publication

Not required.

Data Availability

Data are available upon reasonable request.

For peer review only

References

1. NHS. The NHS Long Term Plan. In: Health Do, ed. Online, 2019.
2. Department of Health and Social Care. NHS Mandate 2018 to 2019 2018 [Available from: https://assets.publishing.service.gov.uk/government/uploads/system/uploads/attachment_data/file/803111/revised-mandate-to-nhs-england-2018-to-2019.pdf accessed 18th August 2019 2019.
3. England PH. Number of coronovirus (COVID-19) cases and risk in the UK 2020 [Available from: https://www.gov.uk/guidance/coronavirus-covid-19-information-for-the-public accessed 29th March 2020.
4. Gilbert A, Billany J, Adam R, et al. Rapid implementation of virtual clinics due to COVID-19: Report and early evaluation of a Quality Improvement initiative. *BMJ Open Quality* 2020;[Accepted ahead of print]
5. WHO. Musculoskeletal conditions fact sheet [Available from: https://www.who.int/mediacentre/factsheets/musculoskeletal/en/ accessed 13th January 2019.
6. Deshpande BR, Katz JN, Solomon DH, et al. Number of Persons With Symptomatic Knee Osteoarthritis in the US: Impact of Race and Ethnicity, Age, Sex, and Obesity. *Arthritis Care and Research* 2016;68(12):1743-50. doi: 10.1002/acr.22897
7. Chris S, Alan AM, Sandra H, et al. Effectiveness of PhysioDirect telephone assessment and advice services for patients with musculoskeletal problems: pragmatic randomised controlled trial. *BMJ: British Medical Journal* 2013;346(7893):14.
8. Donaghy E, Hammersley V, Bikker A, et al. Acceptability, benefits, and challenges of video consulting: A qualitative study in primary care. *British Journal of General Practice* 2019;69(686):E586-E94. doi: 10.3399/bjgp19X704141
9. Armfield NR, Bradford M, Bradford NK. The clinical use of Skype—For which patients, with which problems and in which settings? A snapshot review of the literature. *International Journal of Medical Informatics* 2015;84(10):737-42. doi: 10.1016/j.ijmedinf.2015.06.006
10. Hinman RS, Nelligan RK, Bennell KL, et al. "Sounds a Bit Crazy, But It Was Almost More Personal:" A Qualitative Study of Patient and Clinician Experiences of Physical Therapist-Prescribed Exercise For Knee Osteoarthritis Via Skype. *Arthritis Care & Research* 2017;69(12):1834-44. doi: 10.1002/acr.23218
11. Greenhalgh T, Shaw S, Vijayaraghavan S, et al. Real-world implementation of video outpatient consultations at macro, meso, and micro levels: Mixed-method study. *Journal of Medical Internet Research* 2018;20(4) doi: 10.2196/jmir.9897
12 May C. A rational model for assessing and evaluating complex interventions in health care. *BMC Health Services Research*. 2006;6. doi: 10.1186/1472-6963-6-86
13. May C, Finch, T. Implementing, Embedding, and Integrating Practices: An Outline of Normalization Process Theory. *Sociology* 2009;43(3):535. doi: 10.1177/0038038509103208
14. May C. Towards a general theory of implementation. *Implementation Science* 2013;8(1) doi: 10.1186/1748-5908-8-18
15. May CR, Johnson M, Finch T. Implementation, context and complexity. *Implementation Science.* 2016;11. doi: 10.1186/s13012-016-0506-3

16. Lopriore S, LeCouteur A, Ekberg K, et al. "You'll have to be my eyes and ears": A conversation analytic study of physical examination on a health helpline, 2019:330-39.
17. May CR, Eton DT, Boehmer K, et al. Rethinking the patient: using Burden of Treatment Theory to understand the changing dynamics of illness. *BMC Health Services Research* 2014;14:281-81. doi: 10.1186/1472-6963-14-281
18. Lippiett KA, Richardson A, Myall M, et al. Patients and informal caregivers' experiences of burden of treatment in lung cancer and chronic obstructive pulmonary disease (COPD): a systematic

For peer review only

review and synthesis of qualitative research. *BMJ Open* 2019;9(2):e020515-e15. doi: 10.1136/bmjopen-2017-020515
19. Castle-Clarke S. What will new technology mean for the NHS and its patients? Four big technological trends 2018 [Available from: https://www.nuffieldtrust.org.uk/files/2018-06/1530028974_the-nhs-at-70-what-will-new-technology-mean-for-the-nhs-and-its-patients.pdf.
20. Gilbert AW, Jaggi A, May CR. What is the acceptability of real time 1:1 videoconferencing between clinicians and patients for a follow-up consultation for multi-directional shoulder instability? *Shoulder & Elbow* 2019;11(1):53-59. doi: 10.1177/1758573218796815
21. Hausman DM. Preference, Value, Choice, and Welfare: Cambridge University Press 2012.
22. Hausman DM. Mistakes about Preferences in the Social Sciences, *Philosophy of the Social Sciences.* 2011:3-25. doi: 10.1177/0048393110387885
23 Gilbert AW, Jaggi C, May C, et al. What is the work of being a patient when using communication technology to access healthcare in an orthopaedic setting? A systematic review and meta-synthesis of qualitative studies. PROSPERO CRD42018100896 2018 [Available from: http://www.crd.york.ac.uk/PROSPERO/display_record.php?ID=CRD42018100896.
24. Gilbert A, Jones J, Stokes M, et al. Protocol for the CONNECT Project: a mixed methods study investigating patient preferences for communication technology use in orthopaedic rehabilitation consultations. *BMJ Open* 2019;(Epub ahead of Print) doi: 10.1136/bmjopen-2019-035210
25. CASP. CASP Qualitative Checklist [Available from: https://casp-uk.net/wp-content/uploads/2018/01/CASP-Qualitative-Checklist-2018.pdf accessed 18th June 2019.
26. Harrison R, Macfarlane A, Murray E, et al. Patients' perceptions of joint teleconsultations: a qualitative evaluation. *Health Expectations: An International Journal Of Public Participation In Health Care And Health Policy* 2006;9(1):81-90.
27. Wallace P, Haines A, Harrison R, et al. Joint teleconsultations (virtual outreach) versus standard outpatient appointments for patients referred by their general practitioner for a specialist opinion: a randomised trial. *The Lancet* 2002;359(9322):1961-68. doi: 10.1016/S0140-6736(02)08828-1
28. Young L, Siden H, Tredwell S. Post-surgical telehealth support for children and family care-givers. *Journal of Telemedicine and Telecare* 2007;13(1):15-19. doi: 10.1258/135763307779701149
29. Eriksson L, Lindström B, Ekenberg L. Patients' experiences of telerehabilitation at home after shoulder joint replacement. *Journal Of Telemedicine And Telecare* 2011;17(1):25-30. doi: 10.1258/jtt.2010.100317
30. Cranen K, Drossaert CHC, Brinkman ES, et al. An exploration of chronic pain patients' perceptions of home telerehabilitation services. *Health Expectations* 2012;15(4):339-50. doi: 10.1111/j.1369-7625.2011.00668.x
31. Kairy D, Tousignant M, Leclerc N, et al. The patient's perspective of in-home telerehabilitation physiotherapy services following total knee arthroplasty. *International Journal of Environmental Research and Public Health* 2013;10(9):3998-4011. doi: 10.3390/ijerph10093998
32. Moffett H, Tousignant M, Nadeau S, et al. In-home telerehabilitation compared with face-to-face rehabilitation after total knee arthroplasty. *J Bone Joint Surg Am* 2015;97(14):1129-41.
33. Pearson J, Richardson J, Calnan M, et al. The acceptability to patients of PhysioDirect telephone assessment and advice services; a qualitative interview study. *BMC Health Services Research* 2016;16:104-04. doi: 10.1186/s12913-016-1349-y
34. Salisbury C, Foster NE, Hopper C, et al. A pragmatic randomised controlled trial of the effectiveness and cost-effectiveness of 'PhysioDirect' telephone assessment and advice services for physiotherapy. *Health Technology Assessment (Winchester, England)* 2013;17(2):1. doi: 10.3310/hta17020

For peer review only

35. Dobson F, Hinman RS, French S, et al. Internet-mediated physiotherapy and pain coping skills training for people with persistent knee pain (IMPACT - Knee pain): A randomised controlled trial protocol. *BMC Musculoskeletal Disorders* 2014;15(1) doi: 10.1186/1471-2474-15-279
36. Lawford BJ, Bennell KL, Hinman RS, et al. "I was really sceptical…But it worked really well": a qualitative study of patient perceptions of telephone-delivered exercise therapy by physiotherapists for people with knee osteoarthritis. *Osteoarthritis and Cartilage* 2018;26(6):741-50. doi: 10.1016/j.joca.2018.02.909
37. Hinman RS, Lawford BJ, Campbell PK, et al. Telephone-delivered exercise advice and behavior change support by physical therapists for people with knee osteoarthritis: Protocol for the telecare randomized controlled trial. *Physical Therapy* 2017;97(5):524-36. doi: 10.1093/ptj/pzx021
38. Roberts LC, Whittle CT, Cleland J, et al. Measuring Verbal Communication in Initial Physical Therapy Encounters. *Physical Therapy* 2013;93(4):479-91. doi: 10.2522/ptj.20120089
39. Roberts L, Bucksey SJ. Communicating with patients: what happens in practice? [corrected] [published erratum appears in PHYS THER 2007 Jul;87(7):957]. *Physical Therapy* 2007;87(5):586-94.
40. Katherine JH, Carl RM. Managing expectations: cognitive authority and experienced control in complex healthcare processes. *BMC Health Services Research* 2017(1):1. doi: 10.1186/s12913-017-2366-1
41. Ayo N. Understanding health promotion in a neoliberal climate and the making of health conscious citizens, 2012:99-105.
42. Ellis J, Boger E, Latter S, et al. Conceptualisation of the 'good' self-manager: A qualitative investigation of stakeholder views on the self-management of long-term health conditions. *Social Science & Medicine* 2017;176:25-33. doi: 10.1016/j.socscimed.2017.01.018
43. Kaambwa B, Ratcliffe J, Shulver W, et al. Investigating the preferences of older people for telehealth as a new model of health care service delivery: A discrete choice experiment. *Journal of Telemedicine and Telecare* 2016;23(2):301-13. doi: 10.1177/1357633X16637725
44. Andreassen HK, Dyb K, Pope CJ, et al. Digitized patient–provider interaction: How does it matter? A qualitative meta-synthesis. *Social Science and Medicine* 2018;215:36-44. doi: 10.1016/j.socscimed.2018.08.036
45. Greenhalgh T, Wherton J, Papoutsi C, et al. Beyond adoption: A new framework for theorizing and evaluating nonadoption, abandonment, and challenges to the scale-up, spread, and sustainability of health and care technologies. *Journal of Medical Internet Research* 2017;19(11) doi: 10.2196/jmir.8775
46. Vijayaraghavan S, Morris J, Bhattacharya S, et al. Real-world implementation of video outpatient consultations at macro, meso, and micro levels: Mixed-method study. *Journal of Medical Internet Research* 2018;20(4) doi: 10.2196/jmir.9897
47. Sav A, McMillan SS, Hunter B, et al. Burden of treatment for chronic illness: A concept analysis and review of the literature. *Health Expectations* 2015;18(3):312-24. doi: 10.1111/hex.12046
48. Gibbons CJ, Kenning C, Coventry PA, et al. Development of a Multimorbidity Illness Perceptions Scale (MULTIPleS), 2013.
49. Boyd CM, Wolff JL, Weiss C, et al. Healthcare task difficulty among older adults with multimorbidity. *Medical Care* 2014;52(3 SUPPL. 2):S118-S25. doi: 10.1097/MLR.0b013e3182a977da
50. Tran VT, Barnes C, Ravaud P, et al. Adaptation and validation of the Treatment Burden Questionnaire (TBQ) in English using an internet platform. *BMC Medicine* 2014;12(1) doi: 10.1186/1741-7015-12-109
51. Eton DT, Yost KJ, Lai J-s, et al. Development and validation of the Patient Experience with Treatment and Self-management (PETS): a patient-reported measure of treatment burden, 2017:489-503.

For peer review only

52. Duncan P, Murphy M, Man MS, et al. Development and validation of the Multimorbidity Treatment Burden Questionnaire (MTBQ). *BMJ Open* 2018;8(4) doi: 10.1136/bmjopen-2017-019413
53. Greenhalgh T, Koh GCH, Car J. Covid-19: a remote assessment in primary care. *BMJ (Clinical research ed)* 2020;368:m1182. doi: 10.1136/bmj.m1182
54. Greenhalgh T, Wherton J, Shaw S, et al. Video consultations for covid-19. *BMJ (Clinical research ed)* 2020;368:m998. doi: 10.1136/bmj.m998
55. NHSX. Covid-19 Information Governance advice for health and care professionals 2020 [Available from: https://www.nhsx.nhs.uk/key-information-and-tools/information-governance-guidance/health-care-professionals accessed 29th March 2020 2020.

For peer review only

The CONNECT Project Phase 1 Search Strategy

Searches

English Language studies will be searched for with no date restrictions. The following databases will be searched using a combination of keywords and database specific subject headings: MEDLINE, AMED, CINAHL, PsycINFO and Scopus. Forward and backwards reference screening of identified papers and relevant systematic reviews will be completed using Web of Science.

Types of study to be included

Qualitative studies or studies with a qualitative component (use of interviews or focus groups)

Condition or domain being studied

Orthopaedics and musculoskeletal pathologies

Participants/population

Inclusion:
• Full text academic papers.
• Patients with an orthopaedic / musculosketal problem
• Studies reporting patients accessing clinical care (assessment / rehabilitation) through the use of real time communication technology (eg telephone, videoconferencing) in an orthopaedic / musculoskeletal setting.
• Qualitative studies or studies with a qualitative component (use of interviews or focus groups)

Exclusion:
• Conference abstracts
• Participants without an orthopaedic / musculoskeletal complaint
• Quantitative studies
• Studies not reporting patient viewpoints

Intervention(s), exposure(s)

Inclusion: Participants accessing clinical care (assessment / rehabilitation) through the use of communication technology in real time (eg telephone, videoconferencing) in an orthopaedic / musculoskeletal setting.

Comparator(s)/control

N/A

Context

Studies will be included providing they report the perspectives of patients regarding access to musculoskeletal / orthopaedic assessment / rehabilitation. Studies may also contain perspectives of clinicians in addition to the viewpoints of patients.

Main outcome(s)

Patient experience and workload of using communication technology to access musculoskeletal / orthopaedic assessment / rehabilitation.

Timing and effect measures

Not applicable.

Additional outcome(s)

None

Timing and effect measures

None

Data extraction (selection and coding)

Two researchers will independently identify full text papers meeting the inclusion criteria. Discrepancies arising regarding selection will be resolved through discussion with a third researcher.

Risk of bias (quality) assessment

Two researchers will independently screen the full text included studies for risk of bias. The Critical Appraisal Skills Programme Qualitative Tool will be used. Discrepancies arising regarding risk of bias will be resolved through discussion with a third researcher.

Strategy for data synthesis

All studies included for full text review will be analysed. Data extracted from the Methods, Results, Discussion and Conclusion sections will be analysed by using an attribution analysis. A taxonomy of attributions will be thematically analysed using NVIVO software in accordance with Normalisation Process Theory.

Search Terms

Database	Search Term
MEDLINE	(communication technology OR e-health OR telecare OR telemedicine OR telehealth OR telemonitoring OR videoconferencing OR real time videoconferencing OR real time 1:1 videoconferencing OR telecommunication OR virtual OR (MH "telemedicine") OR (MH "remote consultation") OR (MH "videoconferencing")) AND (Focus group OR Focus groups OR observation OR Ethnography OR Ethnographic OR Phenomenology OR Phenomenological OR Lived experience OR Grounded theory OR Thematic analysis OR Conversation analysis

	OR Framework analysis OR (MH "Qualitative Research")) AND (physio OR physiotherapy OR physical therapy OR rehab OR rehabilitation)
AMED	(communication technology OR e-health OR telecare OR telemedicine OR telehealth OR telemonitoring OR videoconferencing OR real time videoconferencing OR real time 1:1 videoconferencing OR telecommunication OR virtual OR (SU (telemedicine) OR (SU (technology medical))) AND (Focus group OR Focus groups OR observation OR Ethnography OR Ethnographic OR Phenomenology OR Phenomenological OR Lived experience OR Grounded theory OR Thematic analysis OR Conversation analysis OR Framework analysis) AND (physio OR physiotherapy OR physical therapy OR rehab OR rehabilitation)
CINAHL	(communication technology OR e-health OR telecare OR telemedicine OR telehealth OR telemonitoring OR videoconferencing OR real time videoconferencing OR real time 1:1 videoconferencing OR telecommunication OR virtual OR (MH “Videoconferencing”) OR (MH “Remote Consultation”)) AND (Focus group OR Focus groups OR observation OR Ethnography OR Ethnographic OR Phenomenology OR Phenomenological OR Lived experience OR Grounded theory OR Thematic analysis OR Conversation analysis OR Framework analysis OR (MH “qualitative studies”)) AND (physio OR physiotherapy OR physical therapy OR rehab OR rehabilitation)
PsychINFO	(communication technology OR e-health OR telecare OR telemedicine OR telehealth OR telemonitoring OR videoconferencing OR real time videoconferencing OR real time 1:1 videoconferencing OR telecommunication OR virtual OR (DE "Telemedicine")) AND (Focus group OR Focus groups OR observation OR Ethnography OR Ethnographic OR Phenomenology OR Phenomenological OR Lived experience OR Grounded theory OR Thematic analysis OR Conversation analysis OR Framework analysis OR DE "Qualitative Research")) AND (physio OR physiotherapy OR physical therapy OR rehab OR rehabilitation)

SCOPUS	1. (TITLE-ABS-KEY (“communication technology” OR e-health OR telecare OR telemedicine OR telehealth OR telemonitoring OR videoconferencing OR “real time videoconferencing” OR “real time 1:1 videoconferencing” OR telecommunication OR virtual))<br>2. (TITLE-ABS-KEY (“Focus group” OR “Focus groups” OR observation OR Ethnography OR Ethnographic OR Phenomenology OR Phenomenological OR “Lived experience” OR “Grounded theory” OR “Thematic analysis” OR “Conversation analysis” OR “Framework analysis”))<br>3. (TITLE-ABS-KEY (physio OR physiotherapy OR physical therapy OR rehab OR rehabilitation)<br>4. #1 AND #2 AND #3

For peer review only

Figure 1 – PRISMA Flow Diagram of included and excluded studies

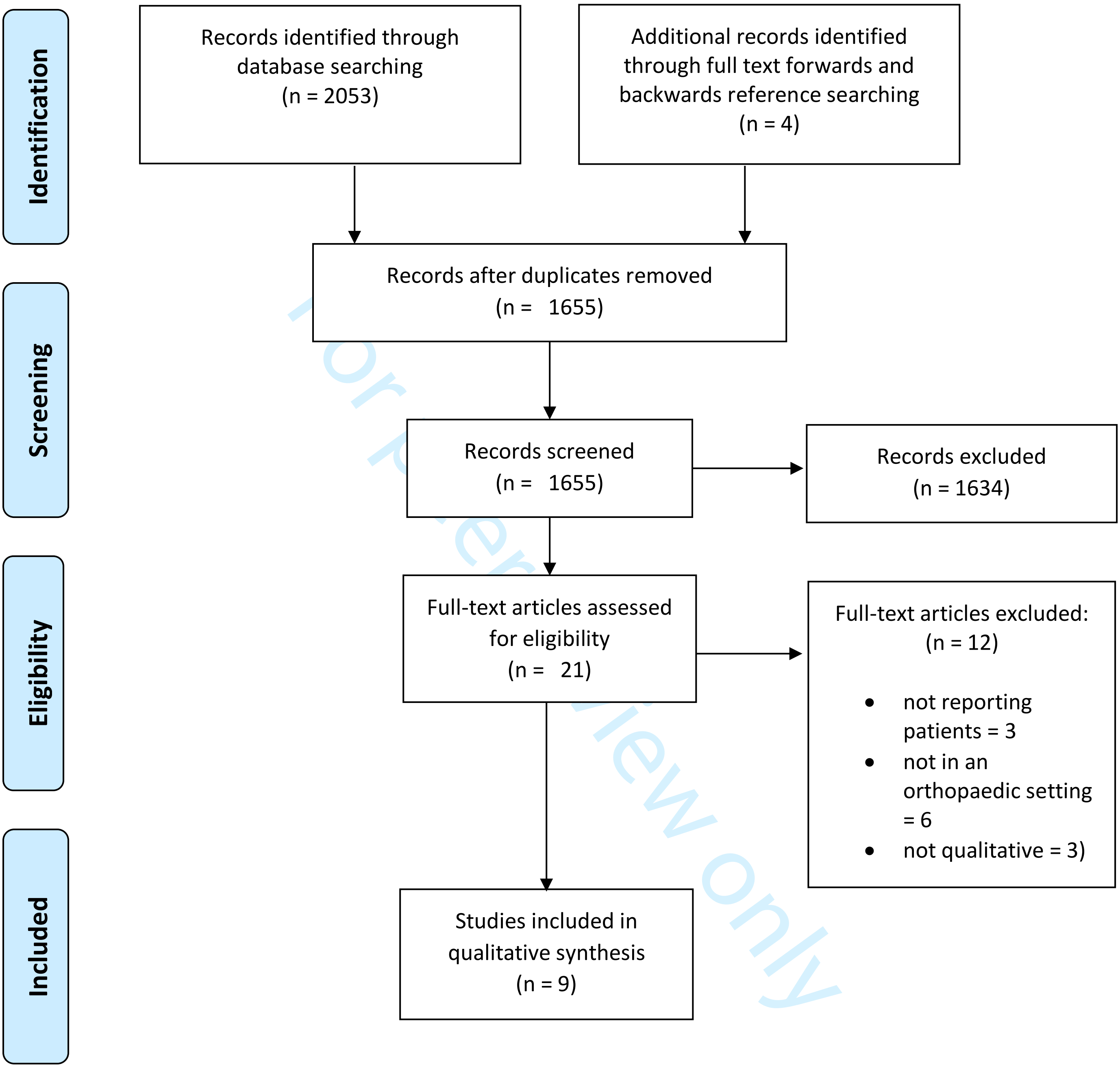

For peer review only

Figure 2 – Visual Model to demonstrate how work influences preference.

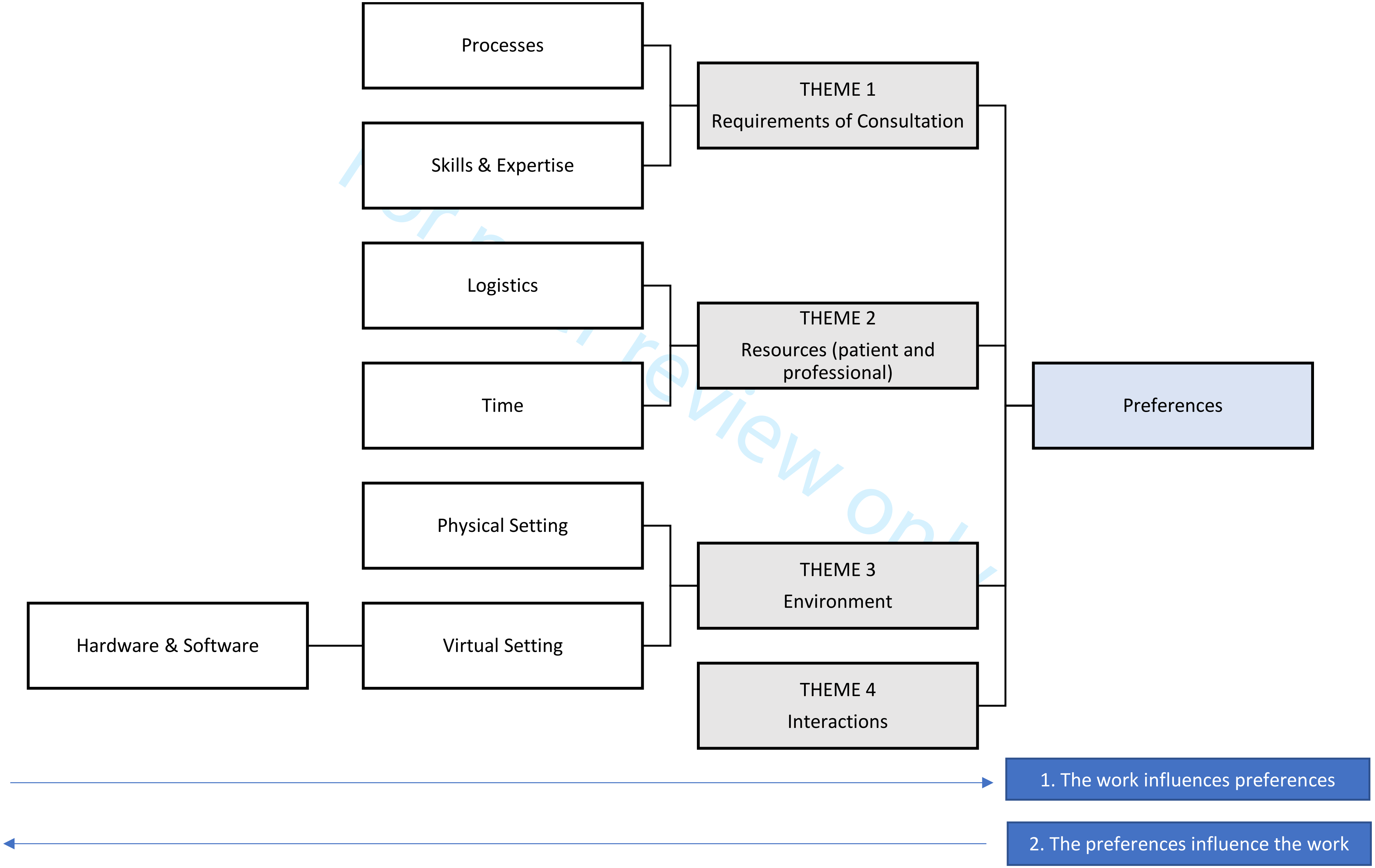

For peer review only - http://bmjopen.bmj.com/site/about/guidelines.xhtml

BMJ Open

The use of virtual consultations for orthopaedics rehabilitation consultations: How do changes in the work of being a patient influence patient preferences? A systematic review and qualitative synthesis

Journal:	*BMJ Open*
Manuscript ID	bmjopen-2019-036197.R2
Article Type:	Original research
Date Submitted by the Author:	25-Jul-2020
Complete List of Authors:	Gilbert, Anthony; Royal National Orthopaedic Hospital Stanmore, Therapies Department; University of Southampton, Faculty of Health Sciences<br>Jones, Jeremy; University of Southampton, Faculty of Health Sciences<br>Jaggi, Anju; Royal National Orthopaedic Hospital Stanmore, Therapies Department<br>May, Carl; London School of Hygiene and Tropical Medicine Faculty of Epidemiology and Population Health
**Primary Subject Heading**:	Qualitative research
Secondary Subject Heading:	Health services research
Keywords:	Telemedicine < BIOTECHNOLOGY & BIOINFORMATICS, ORTHOPAEDIC & TRAUMA SURGERY, QUALITATIVE RESEARCH, REHABILITATION MEDICINE

SCHOLARONE™
Manuscripts

I, the Submitting Author has the right to grant and does grant on behalf of all authors of the Work (as defined in the below author licence), an exclusive licence and/or a non-exclusive licence for contributions from authors who are: i) UK Crown employees; ii) where BMJ has agreed a CC-BY licence shall apply, and/or iii) in accordance with the terms applicable for US Federal Government officers or employees acting as part of their official duties; on a worldwide, perpetual, irrevocable, royalty-free basis to BMJ Publishing Group Ltd ("BMJ") its licensees and where the relevant Journal is co-owned by BMJ to the co-owners of the Journal, to publish the Work in this journal and any other BMJ products and to exploit all rights, as set out in our licence.

The Submitting Author accepts and understands that any supply made under these terms is made by BMJ to the Submitting Author unless you are acting as an employee on behalf of your employer or a postgraduate student of an affiliated institution which is paying any applicable article publishing charge ("APC") for Open Access articles. Where the Submitting Author wishes to make the Work available on an Open Access basis (and intends to pay the relevant APC), the terms of reuse of such Open Access shall be governed by a Creative Commons licence – details of these licences and which Creative Commons licence will apply to this Work are set out in our licence referred to above.

Other than as permitted in any relevant BMJ Author's Self Archiving Policies, I confirm this Work has not been accepted for publication elsewhere, is not being considered for publication elsewhere and does not duplicate material already published. I confirm all authors consent to publication of this Work and authorise the granting of this licence.

For peer review only

The use of virtual consultations for orthopaedics rehabilitation consultations: How do changes in the work of being a patient influence patient preferences? A systematic review and qualitative synthesis

Authors

1. Anthony Gilbert[1, 2]
2. Jeremy Jones[2]
3. Anju Jaggi[1]
4. Carl R May[3]

Author Affiliations

1. Therapies Department, Royal National Orthopaedic Hospital, Stanmore, UK

2. School of Health Sciences, Faculty of Environment and Life Sciences, University of Southampton, Southampton, UK

3. Faculty of Public Health and Policy, London School of Hygiene and Tropical Medicine, London, UK

Corresponding author: Anthony Gilbert anthony.gilbert@nhs.net

Abstract

Objectives

To systematically review qualitative studies reporting the use of virtual consultations within an orthopaedic rehabilitation setting and to understand how it's use changes the work required of patients.

Methods

Following the Preferred Reporting Items for Systematic Reviews and Meta-Analysis Statement we conducted a systematic review of papers to answer the research question 'How do changes in the work of being a patient when using communication technology influence patient preferences?' Electronic databases were searched for studies meeting the inclusion criteria in April 2020.

Results

The search strategy identified 2057 research articles from the database search. A review of titles and abstracts using the inclusion criteria yielded 21 articles for full text review. Nine studies were included in the final analysis. Six studies explored real time videoconferencing and three explored telephone consultations. The use of communication technology changes the work required of patients. Such changes will impact on expectations for care, resources required of patients, the environment of receiving care and patient-clinician interactions. This adjustment of the work required of patients who access orthopaedic rehabilitation using communication technology will

For peer review only

impact on their experience of receiving care. It is proposed that changes in the work of being a patient will influence preferences for or against the use of communication technology consultations for orthopaedic rehabilitation.

Conclusion

We found that the use of communication technology changes the work of being a patient. The change in work required of patients can be both burdensome (it makes it harder for patients to access their care) and beneficial (it makes it easier for patients to access their care). This change will likely to influence preferences. Keeping the concept of patient work at the heart of pathway redesign is likely to be a key consideration to ensure successful implementation.

Registration

PROSPERO registration number: ID=CRD42018100896.

Strength and limitations of this study

- A taxonomy of patient work will assist in understanding implementation processes.
- The use of middle range theory has been employed to guide theorization of the data.
- A secondary analysis of data has been employed to explain concepts which the authors had not originally intended.
- The date range of included studies (2005-2019) include a range of technologies including the use of bespoke software which may present different challenges to modern off the shelf software.

For peer review only

Introduction

Background

The NHS Long Term Plan[1], The United Kingdom's health service's plan to 'make the NHS fit for the future of patients', advocates digital-first primary care and envisions the use of e-consultations to become a new option for every patient. Virtual consultations can support the management of patients with long term conditions such as musculoskeletal disease[2] where long term management may require repeat visits for appointments with healthcare practitioners.

There are examples of virtual consultations in practice. The PhysioDirect telephone and advice service[3] is an example that was found to be safe and resulted in equivalent outcomes to face to face appointments for patients with musculoskeletal disorders. The visual component offered with videoconferencing software offers distinct advantages over telephone consultations[4]. Research has been conducted investigating patients using Skype, a free to access videoconferencing software, to access care[5]. Patients who received telerehabilitation for knee arthritis via SKYPE[6] found it to be feasible and acceptable. The Virtual Online Consultations-Advantages and Limitations (VOCAL) study[7] found video outpatient consultations to be safe, effective and convenient in appropriate situations.

The process of implementing a new intervention (such as the introduction of virtual consultations in healthcare) has been demonstrated to be dependent on how the intervention is operationalised by its users[8], the work people do when they implement a new intervention[9] and the mobilisation of resources over time[10] across different settings[11]. Normalisation Process Theory (NPT) frames implementation processes through its focus on the things people *do* when they implement a new intervention in practice. One study investigated nurse call takers conducting a physical assessment of patients' over a telephone helpline[12]. The study reported nurses' interactions with patients as they instructed them over the phone to perform physical manipulations. The accomplishment of a physical examination required work from patients that differs to face to face consultations. Burden of treatment theory[13] explains how the capacity for action interacts with the work that stems from healthcare. Burden of treatment has been demonstrated to arise when the workload demands exceeds the capacity for patients with COPD and lung cancer[14]. An understanding of the factors that contribute to a change in the work for patients using virtual consultations is an important consideration for patient experience

Research conducted in the UK found that the majority of people say they would use video consultations to consult their General Practitioner about minor ailments and ongoing condition[15]. A proportion (approximately 35%) would not use this modality. Our previous research investigated whether patients preferred face to face or virtual consultations[16]: patients with atraumatic shoulder instability were offered the choice between SKYPE and face to face follow up rehabilitation appointments. Half of patients preferred to see their rehabilitation professional in person[16] in part due to not having access and knowing how to use the software and equipment. The use of SKYPE changed what patients needed to do to engage in their care in our small study and this influenced their choice on whether or not to use it.

Preferences are a set of complex factors that may include enjoyment comparisons *(x to y is preferred if someone enjoys x more than they enjoy y)*, comparative evaluations *(x to y is preferred if someone thinks x is better than y)*, favouring *(selecting x over y because x has a particular set of characteristics)* or choice ranking *(x is chosen over y if and only if they are faced with a choice of x over y)*[17]. To get past the complexities of preferences, preferences can be defined as a 'total

For peer review only

subjective comparative evaluation'[18]. In essence, someone will prefer x over y after consideration of the alternatives, the actions, the state of affairs and the consequences of choosing each alternative. In this paper we are interested in understanding how patient work influences patient preferences.

Aims of this review

This paper reviews qualitative literature on the use of communication technology for patients in an orthopaedic rehabilitation setting to understand how the work of being a patient influences preference. The purpose of this paper is to develop a taxonomy of tasks required of patients using communication technology. We then consider how factors relating to these tasks influence the comparative evaluation patients face when offered the choice of a communication technology or a face to face consultation for orthopaedic rehabilitation.

Methods

A systematic review was conducted using the PRISMA approach in order to answer the research question: *How do changes in the work of being a patient when using virtual consultations influence patient preferences?* This review was registered on the International prospective register of systematic reviews (PROSPERO registration number: ID=CRD42018100896)[19]. The protocol for this review, which forms phase 1 of the CONNECT Project, has previously been published[20].

MEDLINE, AMED, CINAHL, PsychINFO and SCOPUS were searched from inception on the 4th April 2020. Full search terms and the search strategy is available to view in Supplementary Material (See Supplementary Material 1). Articles were screened independently by two authors (AG and AJ) with a third author (JJ) available to discuss any discrepancies (See Figure 1 in Supplementary Material 2 for reporting).

Studies were eligible for inclusion providing they met the criteria for inclusion shown in Table 1. Relevant studies were firstly screened by their title and then by their abstract. Remaining texts were then read in full with all texts retained after this point for qualitative synthesis. Risk of bias was screened using the CASP tool for qualitative studies[21]. A discussion was held, between two authors (AG and AJ) with a third author (JJ) available to discuss any discrepancies, to decide whether included studies were of sufficient quality to include in the review.

Table 1: Eligibility Criteria of Studies

Inclusion:	Exclusion:
• Full text English Language academic papers from inception to 6th April 2020.<br>• Patients with an orthopaedic / musculoskeletal problem<br>• Studies reporting patients accessing physical assessment / rehabilitation through the use of virtual consultations (e.g. telephone, videoconferencing) in an orthopaedic / musculoskeletal setting.<br>• Qualitative studies or studies with a qualitative component that focuses on the patient viewpoint of accessing virtual consultations.	• Conference abstracts<br>• Participants without an orthopaedic / musculoskeletal complaint<br>• Quantitative studies<br>• Studies not reporting patient viewpoints

For peer review only

Full texts were uploaded to QSR NVIVO Software (QSR International Pty Ltd. Version 12, 2018). NVIVO was used to collect and organize data from the results, discussion and conclusion sections of each paper. Each sentence from the included sections were coded on a line by line basis. The codes were labelled using a description of the content of the respective sentence. Data analysis subsequently took three forms: firstly, two authors (AG and CM) conducted a thematic analysis of codes. This was undertaken to familiarize the authors with the content of the papers. For the second iteration of coding the following was considered: *what is the work of being a patient when using virtual consultations?* Codes were then organized into groups depicting the *type* of work required of patients when using virtual consultations to access healthcare. The two authors (AG and CM) then considered the question: *how might the work of being a patient when using virtual consultations influence patient preference?* The data were revisited and theoretical ideas arising from the data were discussed between AG & CM. From here themes, empirical regularities in the data, were identified and characterised. Finally, themes arising from the data were mapped out in the form of a model to demonstrate how, based on the included papers, the change in the work of being a patient might influence preference for virtual consultations.

For peer review only

Results

Study Selection

Systematic search identified 1,655 references (after de-duplication) of which 1,634 were excluded on the basis of titles and abstracts and a further 12 excluded at full text review. As a result 9 papers were included in the review. Of the eight papers, two originated from Australia[6 22], 2 from Canada[23 24] and 3 two from England[16 25 26] and with one from Sweden[27] and one from the Netherlands[28]. Six studies explored real time videoconferencing[6 16 23 24 26-28] and three explored telephone consultations[22 24 25]. Study demographics are shown in Table 2. All studies were screened using the CASP tool for qualitative studies[27] and all were deemed by the authors to be of sufficient quality and therefore retained for analysis.

For peer review only

Table 2 – Study Characteristics.

Included Study	Study Setting	Study Purpose	Technology Used	Participants
Harrison et al 2006[26]	Joint teleconsultations between the patient and their GP and a hospital specialist (England)	To explore patients' experiences of joint teleconferenced consultations	ISDN2 link and off-the-shelf videoconferencing software	28 patients who were enrolled in the Virtual Outreach Randomized Trial[29]. Six patients had a generic orthopaedic diagnosis.
Young et al 2007[24]	Telephone and videophone follow up after scoliosis surgery (Canada)	To better understand the relative effectiveness of two types of telehealth technology, telephone versus videophone, following a child's scoliosis surgery from the perspective of patients and care-givers	For the videophone group patients were provided with a videophone (KXC-AP150, Panasonic, Japan). For the telephone group patients used an ordinary telephone line	43 patients and their families (dyads) who had undergone scoliosis correction surgery. 21 dyads received videophone support and 22 dyads who received telephone support.
Eriksson et al 2011[27]	Video-based physiotherapy at the patient's home for two months after a shoulder replacement (Sweden)	To describe patients' experiences of physiotherapy at home by video-link after a shoulder replacement	Standard commercial videoconferencing units (e.g. Tandberg 800, Sony PCS-50, Polycom VSX 3000)	10 Adults who had undergone a shoulder replacement.
Cranen et al 2011[28]	Telerehabilitation services at a rehabilitation centre (Netherlands)	To explore patients perceptions regarding prospective rehabilitation services and the factors that facilitate or impede patients' intentions to use these services	Home based treatment by means of (unspecified) web cam treatments	25 chronic pain patients from a rehabilitation centre.
Kairy et al 2013[23]	Telerehabilitation between the patient at home and the physical therapist at the hospital (Canada)	To better understand the patient's experience of home telerehabilitation	Internet access and the telerehabilitation platform was installed in the patient's home as reported in[29].	5 patients who had previously received in-home telerehabilitation post knee arthroplasty. Patients were

For peer review only

			The telerehabilitation device was custom built for the study	selected from a pool of participants from the experimental arm of a RCT for in-home telerehabilitation[30].
Pearson et al 2016[25]	Telephone based physiotherapy between a patient and a senior physiotherapist (England)	To describe key variables that determined patient acceptability of the PhysioDirect service and to understand how the patient experience differed from those accessing usual physiotherapy care.	Telephone	57 patients with a musculoskeletal problem. Participants were recruited from the PhysioDirect study[31].
Hinman et al 2017[6]	SKYPE mediated physiotherapy consultations between the patient at home and the physiotherapist (Australia)	To explore the experience of patients and physical therapists with Skype for exercise management of knee osteoarthritis (OA)	SKYPE software	12 patients with a diagnosis of knee osteoarthritis. Participants were key informants from an RCT[32].
Lawford et al 2018[22]	Exercise therapy for people with knee arthritis via telephone (Australia)	To explore people's perceptions of exercise therapy delivered by physiotherapists via telephone.	Telephone	20 patients with knee osteoarthritis. Participants with knee OA were recruited as key informants from an RCT[33].

For peer review only

Gilbert et al 2019[16]	Follow up consultations for patients after a period of inpatient rehabilitation for atraumatic shoulder instability.	To explore reasons behind acceptability of SKYPE follow up consultations	SKYPE software	7 patients chose a SKYPE consultation, 6 patients chose a face to face consultation. In addition, 8 clinicians were interviewed.

For peer review only

Worked Example of Data Analysis

Data from the nine studies were synthesized. All data were treated to the same three step process. An exemplar is demonstrated below using data from Eriksson et al[27]:

1. Data identified (initial line by line identification)

Inability to touch the patient meant therapists were forced to rely more on their subjective assessment of the patient, leading them to spend more time talking with and listening to patients.

2. Data characterized (initial line by line coding)

Code assigned: *Therapists were unable to use 'hands on' during assessment.*

3. Data theorized (consideration of the question: *what is the work of being a patient when using virtual consultations?*)

Patients have to present themselves in a different way during assessment via VC.

Data from the papers are presented in Table 3.

For peer review only

Table 3 - Factors that may affect patient preference for virtual consultations and considerations for virtual consultations

**Finding**	**Construct**	**Results from included papers: factors that contribute towards the work of being a patient when using communication technology**	**Considerations for virtual consultations**
Preferences are shaped by the requirements of the consultation how these change the work	The processes that change	Patients were able to engage in consultation from different places[6]. Using virtual consultations required patients to arrange for additional equipment in the home[23]. They were required to log in to an account[28] and to learn how to use the communication technology[6].	• Consider the impact of changing processes on patients.<br>• Offer troubleshooting for logging in and how to use the equipment.<br>• Consider offering guidance surrounding the suitability of different locations when engaging in virtual consultations.
	The skills & expertise that is required	As patients moved away from physically facilitated exercises there was the requirement to adjust[27], overcome patient-clinician communication difficulties over video-call[24 26] and phone call[25] and face an increased reliance on them to communicate information[6]. In the absence of hands on treatment more emphasis is placed on patients completing exercises[6]. Patients need to self-assess when they cannot be physically assessed by a therapist[6]. Patients may need to adapt to clinicians who do not have adequate communication skills or training for using virtual consultations[22]. Patients may be encouraged to self-monitor improvements more than if they were seen face to face[25].	• Brief and support patients on the changes in style of communication.<br>• Facilitate patients to communicate their problems through a virtual consultation.<br>• Facilitate self-assessment of patients in the absence of clinician's 'hands-on' care.<br>• Facilitate and provide guidance on self-assessment and ongoing monitoring.<br>• Design personalized exercise regimens that are suitable for the patient's clinical problem and their home environment
Preferences are shaped by the resources that are required of patients	Logistics	Use of virtual consultations helps to avoid of transportation issues[6 16 22 23 27 28], reduces travel times[28] for both patients and carers and can increase access to services[6].	• Consider offering virtual consultations for patients who experience difficulty with travel

For peer review only - http://bmjopen.bmj.com/site/about/guidelines.xhtml

	Time	The ease in which exercises can be integrated into home routine[16 28] and through avoidance of travel provides additional time and energy for other activities[27]. Patients valued being able to wait for their appointment in their own chosen environment rather than in the clinic[6 25 26].	• Consider conflicting demands for patients.<br>• Consider the impact of travel and time on patient symptoms.<br>• Consider the impact of patient comfort when waiting for their appointment.
Preferences are shaped by the work required due to the changes in the environment	Setting for physical rehabilitation	Patients had to find ways to overcome a lack of space[6 28] and equipment[6 23] at home. Patients were required to integrate their rehabilitation in the home environment[6 22].	• Support patients to establish a suitable rehabilitation environment at home.<br>• Design treatment regimens based on the patients access to rehabilitation equipment.<br>• Support patients to integrate rehabilitation within the home environment.
	Setting for virtual consultation	At times the rehab was impaired due to technical difficulties[6] and patients felt they missed learning through fellow sufferer contact through not attending the clinic and would need to seek this elsewhere[28].	• Offer troubleshooting when faced with technical difficulties<br>• Consider offering peer support groups for patients who are unable to physically attend the clinic.
	Hardware and software	Patients needed to be supported to access[6 23] and use the equipment[6 16 24 28] and manage to real-time troubleshoot connection problems as they arose[6 24 27 23].	• Consider offering equipment based on the patient's needs.<br>• Tailor support for equipment use based on patient's skill set.<br>• Offer troubleshooting when faced with technical difficulties.
Preferences are shaped by the work that goes into maintaining adequate interactions	Interactions	Patients may have to focus additional attention when communicating over a stutter connection[6 27] or when faced with a language barrier[24]. Patients may need to rely on additional non-verbal communication when communicating over a screen[26]. Patients who feel alienated[28] or detached[25 28] or expect	• Clearly communicate when the connection is impaired; be prepared to abandon and reboot the virtual consultation as required.<br>• Be prepared to emphasize the use of non-verbal communication.

For peer review only

		hands on care[6 24 25] may need to invest additional effort in developing an effective therapeutic the patient clinician relationship.	• Have an awareness of patient preferences; patients who prefer face to face care may require additional input to develop a therapeutic relationship.

For peer review only

Synthesis of Results

Theme 1: Requirements of rehabilitation

1.1 The processes that change

The use of virtual consultations within the treatment pathway required additional steps for patients, such as logging in[28] and setting the software up[6]. Some patients valued the portability of using Skype[6] and found that they could use it across different settings[16] to fulfil the purposes of the consultation. Patients valued the opportunity to run through the processes of using SKYPE for the first time in the form of a 'dummy run'[16].

1.2 The skills & expertise that is required

The use of virtual consultations changed the skills patients needed. Video communication required specific communication skills that included listening with close attention with no interruptions[27]. The gaze of the patients and clinicians were used to signal the start and end of conversations[27]. Patients and their families found it challenging to express how they felt from a distance and were reliant on the visual capabilities of the technology[24]. The lack of visual information was a concern for patients in the PhysioDirect service[25] who did not have visual cues and physical contact. The lack of physical contact meant that therapists were more reliant on information shared by patients rather than those derived from physical tasks[6]. Therapist focussed on more effortful treatments such as exercises and self-management rather than providing them with hands on care[6]Traditional face-to-face interaction is well established and accepted. It was recognised that virtual communication required different skills and therapists' training needs, to ensure effective communication with patients, were considered in one study of telephone consultations[22]. Traditional physiotherapy patient assessment (such as 'hands on' palpation of a joint) is not possible via Skype. As a result of this patients were taught to self-palpate under guidance[6] and instructed how to demonstrate their range of movement over the screen. It is self-evident that visual assessment was not possible over telephone[22 24 25] and this required good communication from both therapists and patients to describe the movements. Patients felt they did not need 'hands on' care when they were seen by an experienced therapist[27] and clinicians were more likely to encourage self-management and exercises when they were seen virtually[25].

Theme 2: Resources

2.1 Logistics

Patients who underwent virtual consultations experienced reduced travel times and transportation issues[6 22 23 27 28] and was often seen as more convenient for patients, particularly those who suffered from chronic pain[6]. Virtual consultations enabled patients to access health services more easily[23 26]. Problems did arise with the PhysioDirect service where patients were unable to get through requiring them having to make multiple calls to speak to a therapist.

2.2 Time

Virtual consultations offered flexibility[22]: *'If I know I'm stuck at work and I can't get to see someone [the telephone] would be a good option...I can ring someone or have an appointment on the phone, and be at work doing what I need to do, and still have my appointment.'* It was particularly useful for patients who had multiple commitments: *'Because life's so busy in general too, so to be able to speak to*

For peer review only

somebody in your home and then you can go on with your, you know, your next thing, is just wonderful...it just opens another brilliant option for people' as it provided more time for other activities and to integrate rehabilitation into daily life[27].

Theme 3: Environment

3.1 Setting for rehabilitation

Rehabilitation in the home was welcomed by some patients as it gave them the opportunity to rehab within their own environment whereas other patients preferred to keep their home environment separate from the clinical environment[28]. Patients found that they had a lack of space at home compared to the clinic[6 28] and could not access clinic-based equipment[6 23]. Rehabilitation required patients to troubleshoot ways to integrate their rehabilitation tasks within the home[6 22].

3.2 Setting for virtual consultation

Some patients valued fellow sufferer contact and felt that through not physically attending the clinic they missed out on stimuli which kept them motivated. Rehabilitation was impaired when there were issues with connectivity and audio-visual interference disrupted the flow of the consultation[6]. Some patients felt that telerehabilitation was as good as real life and didn't affect the flow of the consultation[27].

3.3 Hardware and Software

Patients who did not have access to equipment for virtual consultation needed to be provided with the required hardware[23 24 27]. In some cases, significant support was required for patients to understand how to use the equipment [28 6 24] and to troubleshoot connection problems when they arose[6 23 24 27]. Overcoming these barriers was an important factor in maintaining the quality of the virtual consultation and is likely to require technical support provided by the clinical team[6].

Theme 4: Interactions

Some patients reported being more relaxed in their own home[6]. One patient, however, felt uncertain about having someone looking into their home and aborted the video consultation[27]. Virtual interactions were impaired at times there was a poor connection[6 27] or a language barrier[24]. These situations demanded additional focus and non-verbal communication[26] from the patient. The therapeutic relationship between patients and clinicians is negatively affected when patients feel alienated[28] or detached[25 28] from their clinician. Patients with an expectation of hands on care[6 16 24 25] found virtual rehabilitation more challenging and may need to invest additional effort to maintain an effective relationship with their therapist.

For peer review only

Discussion

This review synthesized nine qualitative studies reporting the use of virtual consultations in an orthopedic setting. We explored how the use of these technologies impacts on the work of being a patient. All studies in this review demonstrated that adjustments are required of patients to operationalise communication technology for virtually mediated clinical interactions. The adjustments (in the work) that a patient needs to make will have an effect on their experiences of receiving care. These experiences, whether previously lived or anticipated in the future, are likely to influence whether or not an individual finds the use of virtual consultations acceptable. The patient preference for a virtual consultation will depend on individual circumstances. Some of these factors which might influence their decision have been and presented in a conceptual model. The model attempts to demonstrate the relationship between patient work and preference when using communication technology. The model suggests that the use of virtual consultations changes the work of being a patient. The consequences (both positive and negative) of these alterations in work may impact on the patient's experience of receiving their healthcare, their burden of treatment and their ability to engage with their healthcare. This is an important consideration for clinician, managers and policy makers.

Clinicians have to pay more attention to the patient as a result of communicating using technology compared to face to face consultations[9]. This appeared to be at odds with traditional consultations where physiotherapists spoke for half of the allotted time compared to patients who spoke for only 33.1%[34] in initial encounters. A study found, during a follow up session between physiotherapists and patients, that physiotherapists spent twice as much time talking as the patients did and they relied on the use of their hands during the session[35]. In addition to the content within sessions, the relationship experienced between the clinician and the patient may differ during a virtual consultation due some patients being more relaxed at home[9].

Some patients expected 'hands-on' treatment. The transfer of clinician manual therapy towards patient self-palpation[6] and exercise[25] may go against what is expected of therapists. The normative expectations of the patients change as a result of the geographical separation (and physical resources that can be mobilized) between patient and therapist[36]. This places particular emphasis on self-management which shifts the responsibility for health away from the state and onto the individual[37]. This is an important consideration as virtual consultations becomes increasingly used in clinical practice. The additional responsibility of self-management[38], the change in work and tasks required to operationalise communication technology may further burden patients as they are rehabilitated virtually.

Patient viewpoints are important. Kaambwa et al[39] found in their study of older people that patients had strong preference for telehealth services that targeted individuals living in remote regions without easy access to clinic. Our previous research[16] demonstrated that distance to travel to a hospital was not the sole reason leading to the acceptability of Skype consultations and that preference is multi-factorial. We found that having rehabilitation in the patient's own environment was preferred by some although bringing the clinical space into the patient's home can change the meaning of their home for them[40]. Greenhalgh et al[41] considers, amongst other things, *what is expected* of the patient when using new technologies and explains that complex tasks are more likely to lead to non-adoption.

Greenhalgh et al's VOCAL study[42] found that the situations where patients were appropriate for video outpatient consultations only formed a fraction of the overall workload. Such situations

For peer review only - http://bmjopen.bmj.com/site/about/guidelines.xhtml

included when close physical examination was not required and when both parties were technically confident and competent. The use of virtual consultations in these situations may increase patient work and therefore contribute towards their burden of treatment. Patients may therefore opt to choose a face to face consultation. Sav et al[43] call for collaborative discussions to help alleviate treatment burden.

Digitally enabled services are a key focus for the UK's National Health Service over the next 10 years[1]. The use of digitally enabled services such as virtual consultations may be useful for some but add to the burden of treatment to others. Tools have been developed to assess burden of treatment[44-48]. Further research investigating the utility of tools such as these may highlight areas where digitally enabled services negatively (or positively) impact on patient experience. The work required and subsequent treatment burden for patients will differ on an individual case by case basis. Table 3 outlines some considerations for clinicians and policymakers considering the use of virtual consultations based on our findings from this systematic review. Further research investigating patient preference will help researchers and clinicians tailor services in a way that suits the need of patients.

Figure 2 in the supplementary material demonstrates how the themes from this review interact with patient preferences. The work required of a patient will influence their expectations of whether or not the use of virtual consultations is acceptable. The logistics and time required of a patient will shape the resources the patient has to dedicate towards their care. The space available and the equipment the patient has access to determines the suitability of the environment. These, coupled with the impact on patient-clinician interactions will determine patient preference for or against virtual consultations. This leads us to our first preposition: Proposition 1: The work required of patients when using virtual consultations will influence their preferences for their use.

Face to face consultations and communication technology consultations have different requirements. Upon choosing a face to face consultation the patient follows the standard pathway. Choosing a communication technology consultation changes what is needed of patients. The change of work demands different skills, processes, expertise, logistical and environmental considerations. This in turn impacts on the nature of the interactions between the patient and their therapist. This leads us to our second preposition: Proposition 2: The preferences regarding the use of virtual consultations will influence the work of being a patient.

The outbreak of 2019 novel coronavirus (COVID 19) was first reported in Wuhan, China and reached the United Kingdom on the 31st January 2020. The COVID-19 virus spreads primarily through droplets of saliva or discharge from the nose when an infected person coughs or sneezes. Social distancing measures have been established with the UK public being placed on 'lockdown' from the 23rd March 2020[49] to avoid transmission of the disease. Healthcare organisations have subsequently embraced the use of virtual consultations to comply with these social distancing measures[50] Th outbreak of COVID-19 has led to a huge upsurge in the interest and importance of virtual consultations in practice[50-52]. As such, many more patients have been forced into undergoing virtual consultations than would have otherwise been required. NHSx recently published information governance advice for health and care professionals [53] to facilitate appropriate use of virtual consultations during COVID-19. Future research should carefully evaluate the consequences of rapid virtual consultation implementation to allow for appropriate redesign of services embracing communication technology. Such redesign should consider how the use of these technologies impact on the work of being a patient.

For peer review only

Limitations of this review

Our review is subject to a number of important limitations. We included papers from the UK, Sweden, USA, Canada, the Netherlands and Australia which utilized a variety of communication technologies. The data that underpins our results are a secondary analysis of other previously collected data. We did not have access to the original qualitative datasets, only that presented in the research papers. To arrive at our conclusions, we have subjected the data from the primary studies to explanatory concepts that the original authors had not intended. The studies spread from 2005 to 2018. During this time technology has advanced considerably and the bespoke software used in the earlier studies (that were developed for the research study) may present different challenges to modern off the shelf software for use with commonly used personal devices such as phones, tablets or computers. It is also important to acknowledge the differences between the different types of technologies. A phone call does not allow for visualization whereas a video call does. Focusing on specific technologies may have generated more applicable results. The original research recruited patients who had opted into these studies. Patients who are satisfied with these technologies are more likely to be recruited to telemedicine studies and may not be a representative sample.

Conclusion

We reviewed eight qualitative studies which reported the use of phone or videocall in orthopaedic care and found that the use of virtual consultations changes the work of being a patient. We identified four different kinds of work relating to: (1) the consultation, (2) the use of resources, (3) changes in the environment and (4) interactions with the healthcare professional. Across all four domains, the change in work required of patients can be both burdensome (it makes it harder for patients to access their care) and beneficial (it makes it easier for patients to access their care). The burden experienced by patients is a result of the relationship between the demands of the work and their capacity to fulfill these demands. Such burden is individual and situational, depending on the clinical requirements and the patient's lifeworld. As a result, we have proposed that the work of being a patient influences their preferences and the resulting choice has consequences on the resulting work that is required of them. Changes in circumstances (such as availability of equipment, understanding of how to use the equipment, requirements of the rehabilitation) may alter what is required both clinically and technologically and influence preferences. This is an important consideration to patients, clinicians, managers and policy makers, especially at a time where the use of technology is being favored during the COVID-19 outbreak. We have demonstrated the importance of considering the work of being a patient when designing and implementing new technologies. Keeping the concept of patient work at the heart of technology implementation is essential to ensure successful uptake in practice.

Patient and Public Involvement

The CONNECT Project Patient and Public Involvement steering group (PPISG) has been set up to provide guidance on the conduct of the research (details available from www.theconnectproject.info). The first meeting of the PPISG was held in August 2016 prior to the submission of the research to the NIHR in May 2017. A discussion was held about the overall research aims which supported the identification of the research questions. The PPISG has supported the design of the overall research plan and will continue to be involved during the development and refinement of each phase prior to the completion of each study protocol. In addition, the PPISG will support the development of the lay-summary outputs to be disseminated to

For peer review only

patients and members of the public. Links to research outputs will be made available on the CONNECT Project website available at www.theconnectproject.info.

Acknowledgements

The authors thank Professor Maria Stokes and members of the CONNECT Project Patient and Public Involvement steering group for their invaluable contributions to the overall study design of the CONNECT Project and obtaining funding for the PhD Fellowship. The authors also thank colleagues within the Therapies Directorate and Research and Innovation Centre at the Royal National Orthopaedic Hospital for their ongoing support.

Contributors

AG wrote the paper and conceived the project with CM and JJ. CRM contributed knowledge on systematic reviews and qualitative analysis. AG and AJ completed the literature search, identification of papers and quality analysis of papers. CM, JJ, and AJ edited and critically revised the paper. All authors have read and approved the manuscript. AG is the guarantor of the manuscript.

Funding Statement

Anthony Gilbert is funded by a National Institute for Health Research (NIHR), Clinical Doctoral Research Fellowship for this research project (ICA-CDRF-2017-03-025).

Disclaimer

This paper presents independent research funded by the National Institute for Health Research (NIHR). The views expressed are those of the authors and not necessarily those of the NHS, the NIHR or the Department of Health and Social Care.

Competing Interests

None Declared

Patient Consent for Publication

Not required.

Data Availability

Data are available upon reasonable request.

For peer review only

References

1. NHS. The NHS Long Term Plan. In: Health Do, ed. Online, 2019.
2. WHO. Musculoskeletal conditions fact sheet [Available from: https://www.who.int/mediacentre/factsheets/musculoskeletal/en/ accessed 13th January 2019.
3. Chris S, Alan AM, Sandra H, et al. Effectiveness of PhysioDirect telephone assessment and advice services for patients with musculoskeletal problems: pragmatic randomised controlled trial. *BMJ: British Medical Journal* 2013;346(7893):14.
4. Donaghy E, Hammersley V, Bikker A, et al. Acceptability, benefits, and challenges of video consulting: A qualitative study in primary care. *British Journal of General Practice* 2019;69(686):E586-E94. doi: 10.3399/bjgp19X704141
5. Armfield NR, Bradford M, Bradford NK. The clinical use of Skype—For which patients, with which problems and in which settings? A snapshot review of the literature. *International Journal of Medical Informatics* 2015;84(10):737-42. doi: 10.1016/j.ijmedinf.2015.06.006
6. Hinman RS, Nelligan RK, Bennell KL, et al. "Sounds a Bit Crazy, But It Was Almost More Personal:" A Qualitative Study of Patient and Clinician Experiences of Physical Therapist-Prescribed Exercise For Knee Osteoarthritis Via Skype. *Arthritis Care & Research* 2017;69(12):1834-44. doi: 10.1002/acr.23218
7. Greenhalgh T, Shaw S, Vijayaraghavan S, et al. Real-world implementation of video outpatient consultations at macro, meso, and micro levels: Mixed-method study. *Journal of Medical Internet Research* 2018;20(4) doi: 10.2196/jmir.9897
8. May C. A rational model for assessing and evaluating complex interventions in health care, 2006.
9. Carl M, Tracy F. Implementing, Embedding, and Integrating Practices: An Outline of Normalization Process Theory. *Sociology* 2009;43(3):535.
10. May C. Towards a general theory of implementation, 2013.
11. May CR, Johnson M, Finch T. Implementation, context and complexity, 2016.
12. Lopriore S, LeCouteur A, Ekberg K, et al. "You'll have to be my eyes and ears": A conversation analytic study of physical examination on a health helpline, 2019:330-39.
13. May CR, Eton DT, Boehmer K, et al. Rethinking the patient: using Burden of Treatment Theory to understand the changing dynamics of illness. *BMC Health Services Research* 2014;14:281-81. doi: 10.1186/1472-6963-14-281
14. Lippiett KA, Richardson A, Myall M, et al. Patients and informal caregivers' experiences of burden of treatment in lung cancer and chronic obstructive pulmonary disease (COPD): a systematic review and synthesis of qualitative research. *BMJ Open* 2019;9(2):e020515-e15. doi: 10.1136/bmjopen-2017-020515
15. Castle-Clarke S. What will new technology mean for the NHS and its patients? Four big technological trends 2018 [Available from: https://www.nuffieldtrust.org.uk/files/2018-06/1530028974_the-nhs-at-70-what-will-new-technology-mean-for-the-nhs-and-its-patients.pdf.
16. Gilbert AW, Jaggi A, May CR. What is the acceptability of real time 1:1 videoconferencing between clinicians and patients for a follow-up consultation for multi-directional shoulder instability? *Shoulder & Elbow* 2019;11(1):53-59. doi: 10.1177/1758573218796815
17. Hausman DM. Preference, Value, Choice, and Welfare: Cambridge University Press 2012.
18. Hausman DM. Mistakes about Preferences in the Social Sciences, 2011:3-25.
19. AW G, A J, C M, et al. What is the work of being a patient when using communication technology

to access healthcare in an orthopaedic setting? A systematic review and

meta-synthesis of qualitative studies. PROSPERO CRD42018100896 2018 [Available from: http://www.crd.york.ac.uk/PROSPERO/display_record.php?ID=CRD42018100896.

For peer review only

20. Gilbert A, Jones J, Stokes M, et al. Protocol for the CONNECT Project: a mixed methods study investigating patient preferences for communication technology use in orthopaedic rehabilitation consultations. *BMJ Open* 2019;(Epub ahead of Print) doi: 10.1136/bmjopen-2019-035210
21. CASP. CASP Qualitative Checklist [Available from: https://casp-uk.net/wp-content/uploads/2018/01/CASP-Qualitative-Checklist-2018.pdf accessed 18th June 2019.
22. Lawford BJ, Bennell KL, Hinman RS, et al. "I was really sceptical...But it worked really well": a qualitative study of patient perceptions of telephone-delivered exercise therapy by physiotherapists for people with knee osteoarthritis. *Osteoarthritis and Cartilage* 2018;26(6):741-50. doi: 10.1016/j.joca.2018.02.909
23. Kairy D, Tousignant M, Leclerc N, et al. The patient's perspective of in-home telerehabilitation physiotherapy services following total knee arthroplasty. *International Journal of Environmental Research and Public Health* 2013;10(9):3998-4011. doi: 10.3390/ijerph10093998
24. Young L, Siden H, Tredwell S. Post-surgical telehealth support for children and family care-givers. *Journal of Telemedicine and Telecare* 2007;13(1):15-19. doi: 10.1258/135763307779701149
25. Pearson J, Richardson J, Calnan M, et al. The acceptability to patients of PhysioDirect telephone assessment and advice services; a qualitative interview study. *BMC Health Services Research* 2016;16:104-04. doi: 10.1186/s12913-016-1349-y
26. Harrison R, Macfarlane A, Murray E, et al. Patients' perceptions of joint teleconsultations: a qualitative evaluation. *Health Expectations: An International Journal Of Public Participation In Health Care And Health Policy* 2006;9(1):81-90.
27. Eriksson L, Lindström B, Ekenberg L. Patients' experiences of telerehabilitation at home after shoulder joint replacement. *Journal Of Telemedicine And Telecare* 2011;17(1):25-30. doi: 10.1258/jtt.2010.100317
28. Cranen K, Drossaert CHC, Brinkman ES, et al. An exploration of chronic pain patients' perceptions of home telerehabilitation services. *Health Expectations* 2012;15(4):339-50. doi: 10.1111/j.1369-7625.2011.00668.x
29. Wallace P, Haines A, Harrison R, et al. Joint teleconsultations (virtual outreach) versus standard outpatient appointments for patients referred by their general practitioner for a specialist opinion: a randomised trial. *The Lancet* 2002;359(9322):1961-68. doi: 10.1016/S0140-6736(02)08828-1
30. Moffett H, Tousignant M, Nadeau S, et al. In-home telerehabilitation compared with face-to-face rehabilitation after total knee arthroplasty. *J Bone Joint Surg Am* 2015;97(14):1129-41.
31. Salisbury C, Foster NE, Hopper C, et al. A pragmatic randomised controlled trial of the effectiveness and cost-effectiveness of 'PhysioDirect' telephone assessment and advice services for physiotherapy. *Health Technology Assessment (Winchester, England)* 2013;17(2):1. doi: 10.3310/hta17020
32. Dobson F, Hinman RS, French S, et al. Internet-mediated physiotherapy and pain coping skills training for people with persistent knee pain (IMPACT - Knee pain): A randomised controlled trial protocol. *BMC Musculoskeletal Disorders* 2014;15(1) doi: 10.1186/1471-2474-15-279
33. Hinman RS, Lawford BJ, Campbell PK, et al. Telephone-delivered exercise advice and behavior change support by physical therapists for people with knee osteoarthritis: Protocol for the telecare randomized controlled trial. *Physical Therapy* 2017;97(5):524-36. doi: 10.1093/ptj/pzx021
34. Roberts LC, Whittle CT, Cleland J, et al. Measuring Verbal Communication in Initial Physical Therapy Encounters. *Physical Therapy* 2013;93(4):479-91. doi: 10.2522/ptj.20120089
35. Roberts L, Bucksey SJ. Communicating with patients: what happens in practice? [corrected] [published erratum appears in PHYS THER 2007 Jul;87(7):957]. *Physical Therapy* 2007;87(5):586-94.

For peer review only

36. Katherine JH, Carl RM. Managing expectations: cognitive authority and experienced control in complex healthcare processes. *BMC Health Services Research* 2017(1):1. doi: 10.1186/s12913-017-2366-1
37. Ayo N. Understanding health promotion in a neoliberal climate and the making of health conscious citizens, 2012:99-105.
38. Ellis J, Boger E, Latter S, et al. Conceptualisation of the 'good' self-manager: A qualitative investigation of stakeholder views on the self-management of long-term health conditions. *Social Science & Medicine* 2017;176:25-33. doi: 10.1016/j.socscimed.2017.01.018
39. Kaambwa B, Ratcliffe J, Shulver W, et al. Investigating the preferences of older people for telehealth as a new model of health care service delivery: A discrete choice experiment. *Journal of Telemedicine and Telecare* 2016;23(2):301-13. doi: 10.1177/1357633X16637725
40. Andreassen HK, Dyb K, Pope CJ, et al. Digitized patient–provider interaction: How does it matter? A qualitative meta-synthesis. *Social Science and Medicine* 2018;215:36-44. doi: 10.1016/j.socscimed.2018.08.036
41. Greenhalgh T, Wherton J, Papoutsi C, et al. Beyond adoption: A new framework for theorizing and evaluating nonadoption, abandonment, and challenges to the scale-up, spread, and sustainability of health and care technologies. *Journal of Medical Internet Research* 2017;19(11) doi: 10.2196/jmir.8775
42. Vijayaraghavan S, Morris J, Bhattacharya S, et al. Real-world implementation of video outpatient consultations at macro, meso, and micro levels: Mixed-method study. *Journal of Medical Internet Research* 2018;20(4) doi: 10.2196/jmir.9897
43. Sav A, McMillan SS, Hunter B, et al. Burden of treatment for chronic illness: A concept analysis and review of the literature. *Health Expectations* 2015;18(3):312-24. doi: 10.1111/hex.12046
44. Gibbons CJ, Kenning C, Coventry PA, et al. Development of a Multimorbidity Illness Perceptions Scale (MULTIPleS), 2013.
45. Boyd CM, Wolff JL, Weiss C, et al. Healthcare task difficulty among older adults with multimorbidity. *Medical Care* 2014;52(3 SUPPL. 2):S118-S25. doi: 10.1097/MLR.0b013e3182a977da
46. Tran VT, Barnes C, Ravaud P, et al. Adaptation and validation of the Treatment Burden Questionnaire (TBQ) in English using an internet platform. *BMC Medicine* 2014;12(1) doi: 10.1186/1741-7015-12-109
47. Eton DT, Yost KJ, Lai J-s, et al. Development and validation of the Patient Experience with Treatment and Self-management (PETS): a patient-reported measure of treatment burden, 2017:489-503.
48. Duncan P, Murphy M, Man MS, et al. Development and validation of the Multimorbidity Treatment Burden Questionnaire (MTBQ). *BMJ Open* 2018;8(4) doi: 10.1136/bmjopen-2017-019413
49. England PH. Number of coronovirus (COVID-19) cases and risk in the UK 2020 [Available from: https://www.gov.uk/guidance/coronavirus-covid-19-information-for-the-public accessed 29th March 2020.
50. Gilbert A, Billany J, Adam R, et al. Rapid implementation of virtual clinics due to COVID-19: Report and early evaluation of a Quality Improvement initiative. *BMJ Open Quality* 2020;[Accepted ahead of print]
51. Greenhalgh T, Koh GCH, Car J. Covid-19: a remote assessment in primary care. *BMJ (Clinical research ed)* 2020;368:m1182. doi: 10.1136/bmj.m1182
52. Greenhalgh T, Wherton J, Shaw S, et al. Video consultations for covid-19. *BMJ (Clinical research ed)* 2020;368:m998. doi: 10.1136/bmj.m998
53. NHSX. Covid-19 Information Governance advice for health and care professionals 2020 [Available from: https://www.nhsx.nhs.uk/key-information-and-tools/information-governance-guidance/health-care-professionals accessed 29th March 2020 2020.

For peer review only

For peer review only

The CONNECT Project Phase 1 Search Strategy

Searches

English Language studies will be searched for with no date restrictions. The following databases will be searched using a combination of keywords and database specific subject headings: MEDLINE, AMED, CINAHL, PsycINFO and Scopus. Forward and backwards reference screening of identified papers and relevant systematic reviews will be completed using Web of Science.

Types of study to be included

Qualitative studies or studies with a qualitative component (use of interviews or focus groups)

Condition or domain being studied

Orthopaedics and musculoskeletal pathologies

Participants/population

Inclusion:
• Full text academic papers.
• Patients with an orthopaedic / musculosketal problem
• Studies reporting patients accessing clinical care (assessment / rehabilitation) through the use of real time communication technology (eg telephone, videoconferencing) in an orthopaedic / musculoskeletal setting.
• Qualitative studies or studies with a qualitative component (use of interviews or focus groups)

Exclusion:
• Conference abstracts
• Participants without an orthopaedic / musculoskeletal complaint
• Quantitative studies
• Studies not reporting patient viewpoints

Intervention(s), exposure(s)

Inclusion: Participants accessing clinical care (assessment / rehabilitation) through the use of communication technology in real time (eg telephone, videoconferencing) in an orthopaedic / musculoskeletal setting.

Comparator(s)/control

N/A

Context

Studies will be included providing they report the perspectives of patients regarding access to musculoskeletal / orthopaedic assessment / rehabilitation. Studies may also contain perspectives of clinicians in addition to the viewpoints of patients.

Main outcome(s)

Patient experience and workload of using communication technology to access musculoskeletal / orthopaedic assessment / rehabilitation.

Timing and effect measures

Not applicable.

Additional outcome(s)

None

Timing and effect measures

None

Data extraction (selection and coding)

Two researchers will independently identify full text papers meeting the inclusion criteria. Discrepancies arising regarding selection will be resolved through discussion with a third researcher.

Risk of bias (quality) assessment

Two researchers will independently screen the full text included studies for risk of bias. The Critical Appraisal Skills Programme Qualitative Tool will be used. Discrepancies arising regarding risk of bias will be resolved through discussion with a third researcher.

Strategy for data synthesis

All studies included for full text review will be analysed. Data extracted from the Methods, Results, Discussion and Conclusion sections will be analysed by using an attribution analysis. A taxonomy of attributions will be thematically analysed using NVIVO software in accordance with Normalisation Process Theory.

Search Terms

Database	Search Term
MEDLINE	(communication technology OR e-health OR telecare OR telemedicine OR telehealth OR telemonitoring OR videoconferencing OR real time videoconferencing OR real time 1:1 videoconferencing OR telecommunication OR virtual OR (MH "telemedicine") OR (MH "remote consultation") OR (MH "videoconferencing")) AND (Focus group OR Focus groups OR observation OR Ethnography OR Ethnographic OR Phenomenology OR Phenomenological OR Lived experience OR Grounded theory OR Thematic analysis OR Conversation analysis

	OR Framework analysis OR (MH "Qualitative Research")) AND (physio OR physiotherapy OR physical therapy OR rehab OR rehabilitation)
AMED	(communication technology OR e-health OR telecare OR telemedicine OR telehealth OR telemonitoring OR videoconferencing OR real time videoconferencing OR real time 1:1 videoconferencing OR telecommunication OR virtual OR (SU (telemedicine) OR (SU (technology medical))) AND (Focus group OR Focus groups OR observation OR Ethnography OR Ethnographic OR Phenomenology OR Phenomenological OR Lived experience OR Grounded theory OR Thematic analysis OR Conversation analysis OR Framework analysis) AND (physio OR physiotherapy OR physical therapy OR rehab OR rehabilitation)
CINAHL	(communication technology OR e-health OR telecare OR telemedicine OR telehealth OR telemonitoring OR videoconferencing OR real time videoconferencing OR real time 1:1 videoconferencing OR telecommunication OR virtual OR (MH “Videoconferencing”) OR (MH “Remote Consultation”)) AND (Focus group OR Focus groups OR observation OR Ethnography OR Ethnographic OR Phenomenology OR Phenomenological OR Lived experience OR Grounded theory OR Thematic analysis OR Conversation analysis OR Framework analysis OR (MH “qualitative studies”)) AND (physio OR physiotherapy OR physical therapy OR rehab OR rehabilitation)
PsychINFO	(communication technology OR e-health OR telecare OR telemedicine OR telehealth OR telemonitoring OR videoconferencing OR real time videoconferencing OR real time 1:1 videoconferencing OR telecommunication OR virtual OR (DE "Telemedicine")) AND (Focus group OR Focus groups OR observation OR Ethnography OR Ethnographic OR Phenomenology OR Phenomenological OR Lived experience OR Grounded theory OR Thematic analysis OR Conversation analysis OR Framework analysis OR DE "Qualitative Research")) AND (physio OR physiotherapy OR physical therapy OR rehab OR rehabilitation)

SCOPUS	1. (TITLE-ABS-KEY (“communication technology” OR e-health OR telecare OR telemedicine OR telehealth OR telemonitoring OR videoconferencing OR “real time videoconferencing” OR “real time 1:1 videoconferencing” OR telecommunication OR virtual))<br>2. (TITLE-ABS-KEY (“Focus group” OR “Focus groups” OR observation OR Ethnography OR Ethnographic OR Phenomenology OR Phenomenological OR “Lived experience” OR “Grounded theory” OR “Thematic analysis” OR “Conversation analysis” OR “Framework analysis”))<br>3. (TITLE-ABS-KEY (physio OR physiotherapy OR physical therapy OR rehab OR rehabilitation)<br>4. #1 AND #2 AND #3

For peer review only

Figure 1 – PRISMA Flow Diagram of included and excluded studies

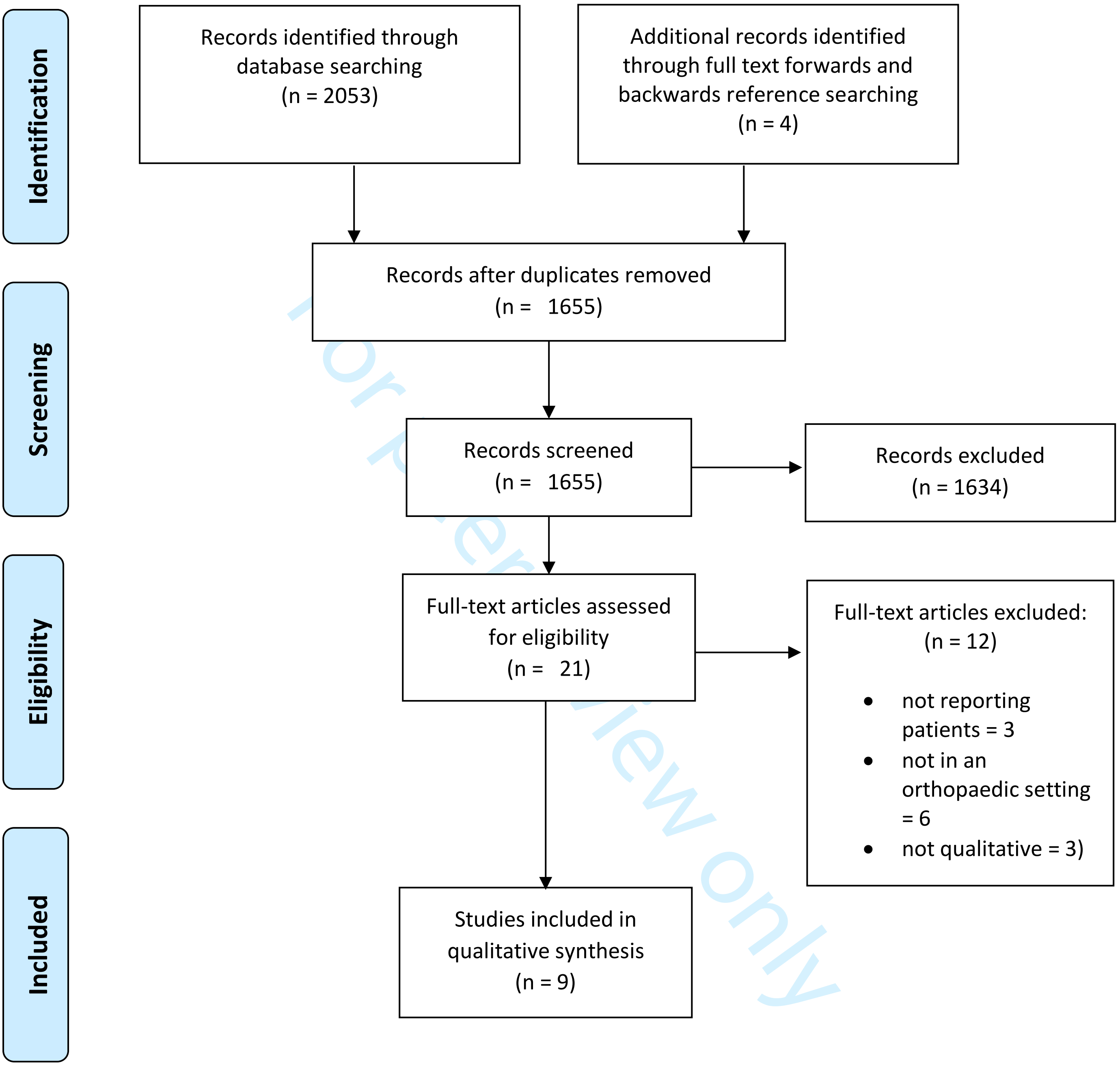

For peer review only - http://bmjopen.bmj.com/site/about/guidelines.xhtml

Figure 2 – Visual Model to demonstrate how work influences preference.

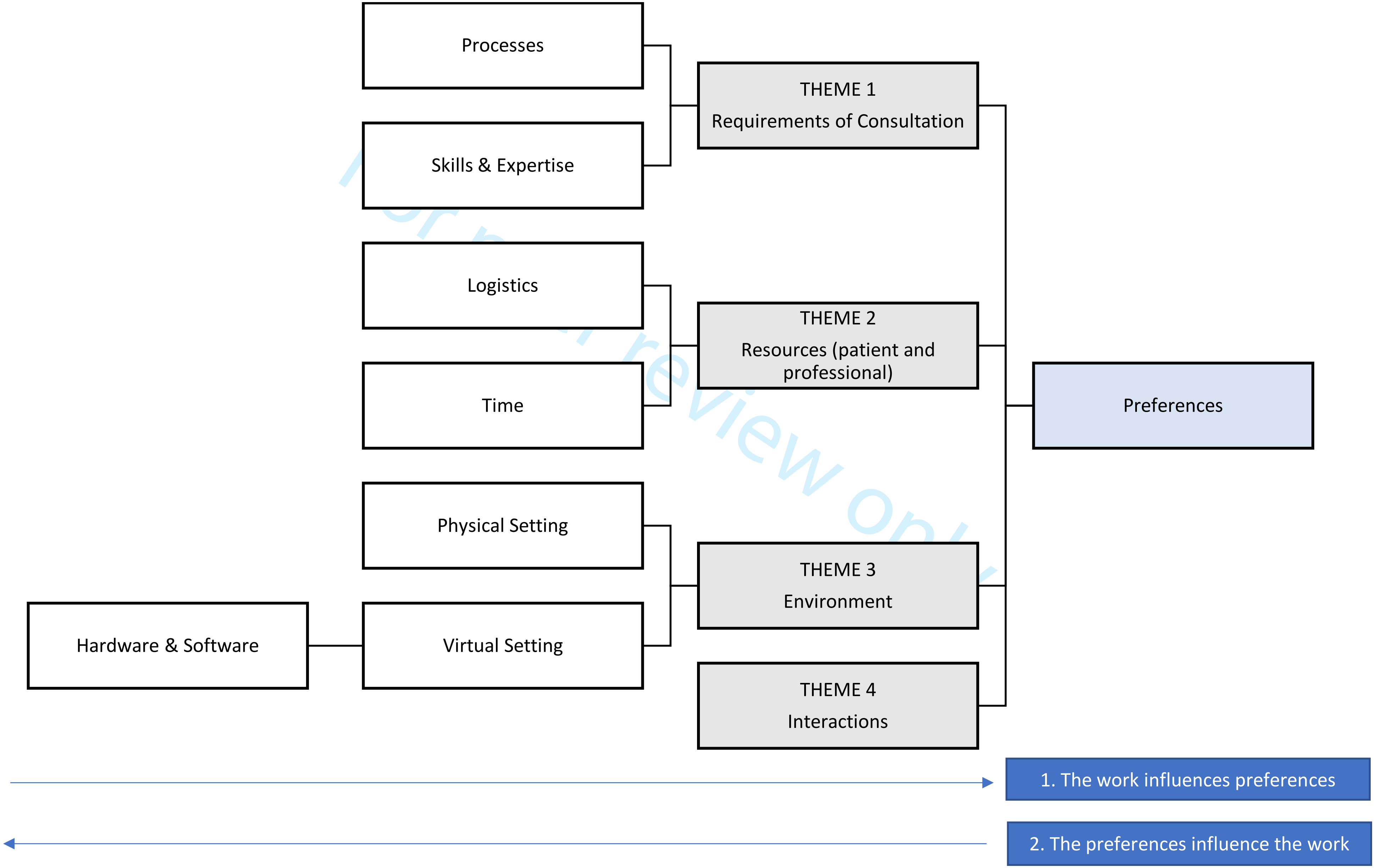

For peer review only - http://bmjopen.bmj.com/site/about/guidelines.xhtml

PRISMA 2009 Checklist

Section/topic	#	Checklist item	Reported on page #
**TITLE**			
Title	1	Identify the report as a systematic review, meta-analysis, or both.	1
**ABSTRACT**			
Structured summary	2	Provide a structured summary including, as applicable: background; objectives; data sources; study eligibility criteria, participants, and interventions; study appraisal and synthesis methods; results; limitations; conclusions and implications of key findings; systematic review registration number.	1
**INTRODUCTION**			
Rationale	3	Describe the rationale for the review in the context of what is already known.	3-4
Objectives	4	Provide an explicit statement of questions being addressed with reference to participants, interventions, comparisons, outcomes, and study design (PICOS).	5
**METHODS**			
Protocol and registration	5	Indicate if a review protocol exists, if and where it can be accessed (e.g., Web address), and, if available, provide registration information including registration number.	4
Eligibility criteria	6	Specify study characteristics (e.g., PICOS, length of follow-up) and report characteristics (e.g., years considered, language, publication status) used as criteria for eligibility, giving rationale.	5
Information sources	7	Describe all information sources (e.g., databases with dates of coverage, contact with study authors to identify additional studies) in the search and date last searched.	4
Search	8	Present full electronic search strategy for at least one database, including any limits used, such that it could be repeated.	Supplementary material 1
Study selection	9	State the process for selecting studies (i.e., screening, eligibility, included in systematic review, and, if applicable, included in the meta-analysis).	4
Data collection process	10	Describe method of data extraction from reports (e.g., piloted forms, independently, in duplicate) and any processes for obtaining and confirming data from investigators.	5
Data items	11	List and define all variables for which data were sought (e.g., PICOS, funding sources) and any assumptions and simplifications made.	5
Risk of bias in individual studies	12	Describe methods used for assessing risk of bias of individual studies (including specification of whether this was done at the study or outcome level), and how this information is to be used in any data synthesis.	4 & 9
Summary measures	13	State the principal summary measures (e.g., risk ratio, difference in means).	9
Synthesis of results	14	Describe the methods of handling data and combining results of studies, if done, including measures of consistency (e.g., I^2) for each meta-analysis.	9

For peer review only

PRISMA 2009 Checklist

Section/topic	#	Checklist item	Reported on page #
Risk of bias across studies	15	Specify any assessment of risk of bias that may affect the cumulative evidence (e.g., publication bias, selective reporting within studies).	9
Additional analyses	16	Describe methods of additional analyses (e.g., sensitivity or subgroup analyses, meta-regression), if done, indicating which were pre-specified.	9
**RESULTS**			
Study selection	17	Give numbers of studies screened, assessed for eligibility, and included in the review, with reasons for exclusions at each stage, ideally with a flow diagram.	6
Study characteristics	18	For each study, present characteristics for which data were extracted (e.g., study size, PICOS, follow-up period) and provide the citations.	7-8
Risk of bias within studies	19	Present data on risk of bias of each study and, if available, any outcome level assessment (see item 12).	9
Results of individual studies	20	For all outcomes considered (benefits or harms), present, for each study: (a) simple summary data for each intervention group (b) effect estimates and confidence intervals, ideally with a forest plot.	n/a
Synthesis of results	21	Present results of each meta-analysis done, including confidence intervals and measures of consistency.	n/a
Risk of bias across studies	22	Present results of any assessment of risk of bias across studies (see Item 15).	9
Additional analysis	23	Give results of additional analyses, if done (e.g., sensitivity or subgroup analyses, meta-regression [see Item 16]).	n/a
**DISCUSSION**			
Summary of evidence	24	Summarize the main findings including the strength of evidence for each main outcome; consider their relevance to key groups (e.g., healthcare providers, users, and policy makers).	13-16
Limitations	25	Discuss limitations at study and outcome level (e.g., risk of bias), and at review-level (e.g., incomplete retrieval of identified research, reporting bias).	16
Conclusions	26	Provide a general interpretation of the results in the context of other evidence, and implications for future research.	16-17
**FUNDING**			
Funding	27	Describe sources of funding for the systematic review and other support (e.g., supply of data); role of funders for the systematic review.	17

From: Moher D, Liberati A, Tetzlaff J, Altman DG, The PRISMA Group (2009). Preferred Reporting Items for Systematic Reviews and Meta-Analyses: The PRISMA Statement. PLoS Med 6(7): e1000097. doi:10.1371/journal.pmed1000097

For more information, visit: www.prisma-statement.org.

For peer review only - http://bmjopen.bmj.com/site/about/guidelines.xhtml
